# Impact of Inoculation Strategy on *Dendrobium nobile* Lindl. Rice Wine Quality Through the Use of *Saccharomyces cerevisiae* FBKL2.8022 and *Wickerhamomyces anomalus* FBKL2.8023

**DOI:** 10.1002/fsn3.72321

**Published:** 2026-09-04

**Authors:** Hongling Gan, Guichun Huang, Lin Zhang, Lanyan Cen, Yifeng Dai, Shuyi Qiu, Xiaodan Wang, Chaoyang Wei

**Affiliations:** ^1^ School of Liquor and Food Engineering Guizhou University Guiyang China; ^2^ Guizhou Key Laboratory of New Quality Processing and Storage of Ecological Specialty Food Guizhou University Guiyang China; ^3^ Development Center of Green Food Guiyang China

**Keywords:** *Dendrobium nobile Lindl.*, flavor substance, metabolites, *Saccharomyces cerevisiae*, *Wickerhamomyces anomalus*

## Abstract

This study examined how the inoculation sequence of *Saccharomyces cerevisiae* (*Sc*) and *Wickerhamomyces anomalus* (*Wa*), along with *Dendrobium nobile* Lindl. (*D. nobile*) addition, affects rice wine quality. Sequential inoculation with *D. nobile* yielded the highest levels of polysaccharides, alkaloids, flavonoids, and phenols. While simultaneous inoculation raised alcohol proportion, sequential inoculation increased ester content. *D. nobile* reduced total flavor concentration but enhanced its diversity. Metabolic analysis indicated that both factors alter metabolites mainly via amino acid and sugar pathways. Thus, sequential *Sc*–*Wa* inoculation with *D. nobile* is an effective strategy for modulating wine composition, providing a reference for improving fermented rice wine quality.

## Graphical abstract

This graphical abstract compares four fermentation strategies for *D. nobile* rice wine: simultaneous inoculation without *D. nobile* (SW), simultaneous inoculation with *D. nobile* (DnSW), sequential inoculation without *D. nobile* (WS), and sequential inoculation with *D. nobile* (DnWS). Simultaneous inoculation (SW/DnSW) enhanced volatile compound content (up to 32.49 mg/L) and alcohol proportion with shorter fermentation (10 days). Sequential inoculation (WS/DnWS) significantly increased functional compounds (polysaccharides, alkaloids, flavonoids, total phenols) and ester diversity, though fermentation was longer (21 days). *D. nobile* addition reduced total flavor concentration but improved flavor diversity. DnWS yielded the highest functional compound content. Metabolic pathways involved were mainly amino acid and carbohydrate metabolism. Sequential inoculation with *D. nobile* is identified as the optimal strategy for functional rice wine production.

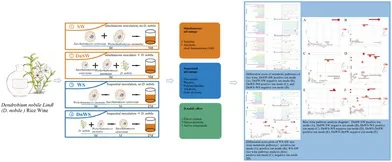

## 1 Introduction

In recent years, in order to enhance the flavor and quality of rice wine, more and more rice wine is fermented with a mixture of different yeast species. Compared with single‐strain fermentation using *Saccharomyces cerevisiae* , mixed fermentation can integrate the metabolic characteristics of different strains, thereby enriching the composition of volatile organic compounds and improving the overall product quality (Huang et al. 2023). Although non‐*Saccharomyces* yeasts are weak in alcoholic fermentation, they play an important role in the quality of fermented wines, especially in aroma characteristics (Luan et al. 2018; Zhou et al. 2021). One of the effects of non‐*Saccharomyces* yeasts on wine characteristics is their enzymes and metabolites, and another is the formation of volatile flavor compounds as a result of metabolism (Fang et al. 2019). In the study by Huang et al. (Huang et al. 2023), mixed fermentation of *Pichia kudriavzevii* with *Saccharomyces cerevisiae* could effectively integrate the different characteristics and advantages of the strains, that is increase the amount of volatile compounds and appropriately lengthen the fermentation time, to enrich the flavor and improve the quality of rice wine. The ability of *Saccharomyces cerevisiae* to rapidly consume nitrogen and carbon sources in the fermentation system, coupled with high ethanol tolerance, gives them a key adaptive advantage under abiotic stress conditions during alcoholic wine fermentation, at the expense of non‐Saccharomyces yeast species (Comitini et al. 2021).

However, the combination of different yeast strains and inoculation strategies (simultaneous vs. sequential inoculation) have significant effects on fermentation efficiency, aroma generation, and sensory properties. Simultaneous inoculation means that non‐ *Saccharomyces cerevisiae* and *Saccharomyces cerevisiae* are inoculated for fermentation at the same time, while sequential inoculation means that a non‐*Saccharomyces* yeast is inoculated before *Saccharomyces cerevisiae* . The core advantage of simultaneous inoculation lies in shortening the production cycle and simplifying process operations. For example, in wine malolactic fermentation, simultaneous inoculation of lactic acid bacteria with yeast can shorten the total fermentation time, avoiding the inhibition of lactic acid bacteria caused by the high‐ethanol environment in traditional sequential inoculation (Zhao et al. 2022). However, simultaneous inoculation has obvious limitations in terms of aroma quality. Studies have shown that, compared to sequential inoculation, the concentrations of key fruity esters (such as isoamyl acetate and ethyl octanoate) in strawberry wines produced by simultaneous inoculation were significantly lower, accompanied by lower sensory evaluation scores (Zhang et al. 2024). Similarly, studies on bilberry wine have shown that although simultaneous inoculation can reduce some off‐flavors caused by pure fermentation of non‐*Saccharomyces* yeasts, its promoting effect on esters and higher alcohols is weaker than that of sequential inoculation (Liu et al. 2018; Liu et al. 2019). Sequential inoculation, on the other hand, exhibits significant advantages in improving aroma quality and sensory characteristics. Inoculating non‐yeasts first favors their metabolic activity, either through low production of ethanol, volatile acids or enhancement of specific aromatic compounds (Canonico et al. 2021). In strawberry wine, sequential inoculation of *Torulaspora delbrueckii* and *Saccharomyces cerevisiae* significantly increased the concentrations of fruity esters and higher alcohols, resulting in higher sensory scores for “fruity” and “floral” notes (Zhang et al. 2024). Studies on bilberry wine further confirmed that, regardless of whether *Torulaspora delbrueckii* or *Schizosaccharomyces* pombe was used, sequential inoculation effectively integrated the positive characteristics of non‐*Saccharomyces* yeasts, such as low ethanol yield and high production of fruity esters, while avoiding off‐flavors (e.g., excessive acetaldehyde or acetoin) produced during their pure fermentation (Liu et al. 2018, 2019). It has been reported that sequential inoculation with non‐*Saccharomyces* yeasts and *Saccharomyces cerevisiae* increased the medium chain fatty acid ethyl ester content compared to simultaneous co‐fermentation (Hu et al. 2018). Moreover, sequential inoculation can also regulate acidity, activate amino acid metabolism, and enhance the umami and richness of the product (Hu et al. 2018; Li et al. 2025). However, sequential inoculation still faces challenges in practical applications: the early growth of non‐Saccharomyces yeasts may compete for nutrients with the subsequently inoculated *Saccharomyces cerevisiae* , affecting fermentation efficiency and stability. Meanwhile, the metabolic interactions between different yeast species remain unclear, leading to a lack of systematic guidance for optimizing inoculation ratios and time intervals.

*Dendrobium nobile* Lindl. is a medicinal plant rich in many bio‐active compounds (Li et al. 2024). *Dendrobium* alkaloids, polysaccharides and flavonoids in *D. nobile* have anti‐fatigue, immunomodulatory and neuroprotective effects (Li et al. 2022). Adding medicinal ingredients to the fermentation of rice wine not only enriches the color and flavor of the wine, but also brings nutritional value and health benefits (Lou et al. 2023). For example, sweet rice wine fortified with *Eucommia ulmoides* leaves exhibits strong antioxidant activity as well as in vitro antihypertensive, hypoglycemic, and hypolipidemic effects (Ren et al. 2023). These studies indicate that incorporating medicinal plants into the fermentation system is an effective approach for developing functional rice wine. However, existing studies on *D. nobile* rice wine have predominantly concentrated on single‐strain fermentation, while systematic investigations employing mixed cultures, particularly sequential inoculation strategies, remain scarce.

In the pre‐laboratory stage, excellent strains with low yield of higher alcohols, *Saccharomyces cerevisiae* FBKL2.8022 and *Wickerhamomyces anomalus* FBKL2.8023, were isolated and screened from the traditional Guizhou lumpy koji, but their application to the mixed fermentation of rice wine has not been reported. In view of this, we used *Saccharomyces cerevisiae* FBKL2.8022 (*Sc*) and *Wickerhamomyces anomalus* FBKL2.8023 (*Wa*) for the mixed fermentation of *D. nobile* rice wine. The physicochemical parameters, organoleptic properties, active compounds, and volatile flavor compounds in the fermented *D. nobile* rice wine, as well as the identification of the main differential metabolites and metabolic pathways in combination with metabolomics, were investigated. The aim was to preliminarily investigate the difference between different inoculation strategies of fermentation in mixed fermentation of *Sc* and *Wa*, and the effect of *D. nobile* on fermented rice wine, to provide a theoretical basis for the research and development of *D. nobile* products, as well as its application in fermented rice wine.

## 2 Materials and Methods

### 2.1 Microorganisms

*Saccharomyces cerevisiae* FBKL2.8022 and *Wickerhamomyces anomalus* FBKL2.8023 were isolated, screened and identified for preservation from Guizhou traditional Chinese Xiaoqu by the Guizhou Provincial Key Laboratory of Fermentation Engineering and Biopharmaceuticals with the preservation numbers of CCTCC NO M2019406 and M2019412. *D. nobile* was purchased from Guizhou Chishui Guoli Dendrobium Development Co. Ltd. (Chishui, China).

### 2.2 Rice Wine Fermentation

Rice wine was fermented using a mixture of *Sc* and *Wa*. The preserved *Sc* and *Wa* were incubated in PDA medium (potato dextrose agar medium) at 30°C for 48 h. Single colonies with good growth were selected and inoculated in wort medium and incubated on a shaker at 110 rpm/min at 30°C for 12 h. Glutinous rice was soaked in 2.5 times the mass of water overnight, drained, and steamed at 121°C for 40 min. Glutinous rice was purchased from Dalian Dehexin Agricultural Products Processing Co. Ltd. (Dalian, China). Saccharase, amylase and sterile water with 0.56%, 1.89% and 150% of the mass of glutinous rice were added, mixed well, and placed in water bath at 60°C for 30 min. *D. nobile* powder (passed through a 40‐mesh sieve) was added at a concentration of 2% (*w*/*w*) based on the mass of glutinous rice. The *Sc* and *Wa* were inoculated at a total concentration of 1 × 10^6^ cells/mL with a cell number ratio of *Sc*:*Wa* = 10:1, and the fermentation was carried out at a constant temperature of 30°C, and the fermentation was carried out at a constant temperature of 30°C. The fermentation broth was centrifuged at 8000 r/min for 10 min and the supernatant was taken to obtain the fermented rice wine.

Preliminary optimization of the *Sc* and *Wa* inoculation ratios, inoculation sequence, and *D. nobile* additions was carried out, respectively (Supporting Information, abbreviated as S1). Based on the results of the preliminary experiments, fermentation was carried out with *Sc* and *Wa* under optimal fermentation conditions. Four different fermentations were included: simultaneous inoculation of *Sc* and *Wa* as SW and addition of *D. nobile* in SW as DnSW. Sequential inoculation of *Sc* and *Wa* as WS and addition of *D. nobile* in WS as DnWS.

For sequential inoculation (WS and DnWS), Wa was inoculated first at the same concentration as in the simultaneous group and fermented alone for 5 days; then on day 5, Sc was added at the same concentration as in the simultaneous group.

### 2.3 Physicochemical Determination of Rice Wine

The fermentation broth was taken and filtered through a 0.45‐μm filter membrane. Alcohol content was determined using a biosensor analyzer (Silman Technology Co., Shenzhen, China) (Yao et al. 2023). The total sugars were determined by the phenol‐sulfuric acid method (Albalasmeh et al. 2013). Reducing sugars were determined by 3,5‐Dinitrosalicylic acid (Mercado‐Pacheco et al. 2020). The total acid content was determined with reference to GB/T 13662‐2018 “Yellow Wine” by pH potential method. The pH was determined using a precision pH meter PSH‐3C (Shanghai Nissima Scientific Instruments Co., Shanghai, China).

### 2.4 Sensory Evaluation

Sensory evaluation was referred to T/GZSX 017‐2020 “Guizhou Rice Wine” and GB/T 13662‐2018 “Yellow Wine” with slight modification. Ten professionals were selected for sensory evaluation in terms of color and appearance, aroma, flavor, and style. All participants involved in the sensory evaluation provided written informed consent prior to the study. Samples were coded with random three‐digit numbers and presented in uniform tasting glasses. Blind tasting was conducted at room temperature (22°C ± 2°C). After evaluating each sample, panelists rinsed their mouths with purified water and waited for 2 min before evaluating the next sample. The rice wine was evaluated for color and appearance, aroma, taste, and overall style, with each attribute scored on a 25‐point scale (total possible score 100 points). The scoring criteria are presented in Table S1.

### 2.5 Determination of the Content of Active Compounds

The polysaccharides in the fermentation broth were extracted and the content was determined by phenol‐sulfuric acid method (Albalasmeh et al. 2013). Flavonoid content was determined according to Escudero‐Lopez et al. (2013). Total phenol content was determined by Folin–Ciocalteu method (Tounsi et al. 2011). *Dendrobium* alkaloid content was determined by referring to the method of Wang et al. (2016) with slight modification.

### 2.6 Determination of Volatile Flavor Compounds

The volatile components of the fermentation broth were determined according to the method of Cen et al. (2023). The volatile components in rice wine were determined using headspace solid‐phase microextraction coupled with gas chromatography mass spectrometry (SPME–GC–MS) using a 7890A–5975C gas chromatograph mass spectrometer (Agilent Technologies Inc., Santa Clara, CA, USA). A total of 5.0 mL of the rice wine mixture was added to a 20‐mL extraction bottle, followed by the addition of 0.2 g of NaCl and 40 μL of 2‐octanol (0.18 mg/mL) as an internal standard. The mixture was shaken well and sealed, and then take subjected to a water bath at 60°C for 10 min for Hheat preservation. Afterward, headspace extraction was carried out for 30 min, followed by sample injection and desorption for 3 min. The chromatographic conditions included a DB–Wax column (30 m × 0.25 mm, 0.25 μm), split‐free mode, column box temperature of 40°C, inlet temperature of 250°C and helium (He) flow rate of 1.00 mL/min. The heating program involved maintaining the initial column temperature at 40°C for 1 min, increasing to 180°C at 5°C/min for 1 min and then reaching 230°C at 8°C/min for 7 min.

### 2.7 Determination of Metabolites

The extraction and determination of metabolites were referred to the method of Cen et al. (Cen et al. 2023). Extraction of metabolites: a total of 50 μL of the sample was transferred to an EP tube. After the addition of 200 μL of extract solution (acetonitrile:methanol = 1:1, containing an isotopically labeled internal standard mixture), the samples were vortexed for 30 s, sonicated for 10 min in an ice‐water bath and incubated for 1 h at −40°C to precipitate the proteins. The sample was then centrifuged at 12,000 rpm (RCF = 13,800× *g*, *R* = 8.6 cm) for 15 min at 4°C. The resulting supernatant was transferred to a fresh glass vial for analysis. The quality control (QC) sample was prepared by mixing equal amounts of supernatants from all the samples.

LC–MS/MS analyses were performed using a UHPLC system (Vanquish, Thermo Fisher Scientific, Waltham, MA, USA) with a UPLC BEH Amide column (2.1 mm × 100 mm, 1.7 μm) coupled to a Q Exactive HFX mass spectrometer (Orbitrap MS, Thermo). Mobile phase A was an aqueous solution of 25 mmol/L ammonium acetate and 25 mmol/L ammonium hydroxide (pH = 9.75), and mobile phase B was acetonitrile. The auto‐sampler temperature was 4°C, and the injection volume was 2 μL.

A QE HFX mass spectrometer was used for its ability to acquire MS/MS spectra on information‐dependent acquisition (IDA) mode in the control of the acquisition software (Xcalibur, Thermo). In this mode, the acquisition software continuously evaluates the full MS spectrum scan. The ESI source conditions were established as follows: the sheath gas flow rate was 30 Arb, the Au gas flow rate was 25 Arb, the capillary temperature was 350°C, full MS resolution was 60,000, MS/MS resolution was 7500, collision energy was 10/30/60 in NCE mode, and the spray voltage was 3.6 kV (positive) or −3.2 kV (negative), respectively.

### 2.8 Statistical Analysis

All experiments were set up with 3 replicates and the results were expressed as mean ± standard deviation. Data were analyzed for significance of differences using IBM SPSS Statistics version 26.0. Drawing graphics using Adobe Illustrator 2020, Origin 2021, ProteoWizard, and so forth.

For metabolomics data: raw data were converted with ProteoWizard, processed using XCMS‐based R software, and annotated against the BiotreeDB database (cutoff 0.3). Peaks detected in < 50% of QC samples were removed; missing values were imputed with half of the minimum value. Data were normalized by TIC, log‐transformed, and Pareto‐scaled. PCA and OPLS‐DA were performed using SIMCA 16.0.2, and models were validated by 200‐fold permutation tests. Differentially accumulated metabolites were identified using VIP > 1.0 and *p* < 0.05 (Student's *t*‐test). Pathway enrichment was performed with the KEGG database (reference species *Saccharomyces cerevisiae* ) using the hypergeometric test and Benjamini–Hochberg correction (*Q* < 0.05).

## 3 Results and Discussion

### 3.1 Analysis of Quality of Rice Wine

#### 3.1.1 Physicochemical Indicators

The optimized fermentation process for the rice wine was as follows: the inoculation ratio of *Sc* and *Wa* was 10:1, and the addition of *D. nobile* was 2% of the mass of glutinous rice. The sequential inoculation condition was *Wa* for individual fermentation first, followed by *Sc* on the 5th day (S1).

During optimization, the Sc:Wa ratio (1:1 to 60:1) and *D. nobile* addition (1%–5%) did not affect the fermentation cycle, as all groups reached endpoint on day 10 (Figure S1A,B). However, inoculation sequence significantly influenced fermentation duration: earlier Sc addition (day 0–1) resulted in day‐10 completion with higher CO_2_ weight loss, while delayed Sc addition (after day 4) extended the period to day 23 with lower weight loss (Figure S1C). Based on physicochemical analysis, the optimal *Sc*:*Wa* ratio of 10:1 was selected (Figure S2); 2% *D. nobile* addition gave the best balance of active components and sensory quality (Figure S3), and *Wa* first with *Sc* added on day 5 was chosen as the optimal sequence (Figure S4).

The change in CO_2_ weight loss of rice wine during the fermentation of the four fermentation methods was shown in Figure S1D. Simultaneous inoculation reached the end of fermentation on the 10th day, and sequential inoculation reached the end of fermentation on the 21st day. There were larger differences between the fermentation period and the change in CO_2_ weight loss during fermentation for rice wine fermented by different inoculation methods of *Sc* and *Wa*. Sequential inoculation fermentation resulted in a longer fermentation period for rice wine, with *Wa* as the dominant yeast in the fermentation system, and the change in CO_2_ weight loss was more gentle. The change in weight loss of rice wine fermented by simultaneous inoculation was more pronounced, suggesting that simultaneous inoculation of *Sc* and *Wa* could be effective for alcoholic fermentation. Alcoholic fermentation was larger inhibited in the sequentially inoculated fermentation system, so it was hypothesized that *Wa* and its metabolites in sequentially inoculated fermentation might have an inhibitory effect on *Sc*.

The pH and alcohol content of simultaneously inoculated rice wines were significantly higher than those of sequentially inoculated rice wine, and the total sugars, reducing sugars and total acids were significantly lower than those of sequentially inoculated rice wine (Table 1). This indicated that *Sc* dominated the fermentation in the simultaneous inoculated fermentations and had high efficiency in converting sugars into ethanol. In sequential inoculation fermentation, *Wa* occupied a dominant fermentation role, and the fermentation had a lower utilization rate for the conversion of saccharides and a weak alcoholic fermentation capacity compared to the simultaneous inoculated fermentations. There were no significant differences in total sugar, reducing sugar, alcohol, and sensory scores in DnSW and SW, indicating that the addition of *D. nobile* did not have a significant effect on the rice wine fermented by simultaneous inoculation of *Sc* and *Wa*. Reducing sugar and total acid content of DnWS rice wines were significantly higher than those of WS and the total sugar was significantly lower in the DnWS wines than in the WS wines. This indicated that *D. nobile* had a significant effect on rice wine fermented by sequential inoculation of *Sc* and *Wa*. The inoculation sequence of *Sc* and *Wa* had a significant effect on the physicochemical parameters of rice wines, while the addition of *D. nobile* had a lesser effect.

**TABLE 1:** Physicochemical parameters of rice wine produced with different inoculation strategies.

	Total sugar (mg/mL)	Reducing sugar (mg/mL)	Total acid (mg/mL)	pH	Alcohol content (%vol)	Sensory score
SW	60.06 ± 1.34^c^	36.13 ± 0.81^c^	1.78 ± 0.01^d^	4.31 ± 0.01^b^	24.05 ± 0.00^a^	70.00 ± 2.65^a^
DnSW	60.96 ± 1.04^c^	37.04 ± 2.06^c^	1.80 ± 0.01^c^	4.36 ± 0.03^a^	23.68 ± 0.98^a^	73.33 ± 2.31^a^
WS	118.54 ± 6.21^a^	81.86 ± 2.08^b^	3.85 ± 0.01^b^	3.98 ± 0.02^c^	18.00 ± 1.08^b^	69.67 ± 1.53^a^
DnWS	108.04 ± 3.60^b^	88.76 ± 2.51^a^	3.88 ± 0.01^a^	4.00 ± 0.01^c^	16.40 ± 0.94^b^	71.00 ± 4.58^a^

*Note:* Different lowercase letters in the same column indicate significant differences, *p* < 0.05. Simultaneous inoculation of *Sc* and *Wa* as SW and addition of *D. nobile* in SW as DnSW. Sequential inoculation of *Sc* and *Wa* as WS and addition of *D. nobile* in WS as DnWS.

#### 3.1.2 Analysis of Active Compounds

The content of the active compounds in the four rice wines are shown in Table 2. The content of polysaccharides, total phenols and other active compounds in DnWS and WS were significantly higher than those in DnSW and SW, indicating that sequential inoculation fermentation of *Wa* and *Sc* was favorable to the leaching of active compounds (Liu et al. 2023). The content of *Dendrobium* alkaloid and flavonoid in DnWS were higher than those of other rice wine, indicating that sequential inoculation better promotes the metabolic transformation of active compounds in the fermentation system. The content of the actives in the rice wine after fermentation with the addition of *D. nobile* all showed an increasing trend, and it was speculated that the increased active compounds were mainly derived from *D. nobile* or from the metabolic transformation of *D. nobile* by *Sc* and *Wa*. Sequential inoculated fermentations can better support the release of active compounds and metabolic transformation in *D. nobile*. The *Dendrobium* alkaloid content in DnWS was much higher than that in DnSW, indicating that sequential inoculated fermentations had the most significant effect on the *Dendrobium* alkaloid content in rice wine.

**TABLE 2:** Content of active compounds in rice wine.

	Polysaccharides (mg/mL)	*Dendrobium* alkaloid (PPM)	Flavonoids (μg/mL)	Total phenols (mg/mL)
SW	2.03 ± 0.11^c^	—	35.40 ± 0.010^d^	0.37 ± 0.004^d^
DnSW	2.48 ± 0.17^c^	12.17 ± 1.72^b^	52.04 ± 0.001^b^	0.44 ± 0.006^c^
WS	3.61 ± 0.33^b^	—	43.18 ± 0.001^c^	0.57 ± 0.004^b^
DnWS	4.83 ± 0.33^a^	35.45 ± 0.24^a^	62.83 ± 0.002^a^	0.61 ± 0.019^a^

*Note:* Different lowercase letters in the same column indicate significant differences, *p* < 0.05.

#### 3.1.3 Analysis of Volatile Flavor Compounds

Some studies have shown that mixed fermentation of *Saccharomyces cerevisiae* with non‐*Saccharomyces* yeasts can increase the volatile aroma components in wine (Xu et al. 2022). The dominant strain during fermentation largely determines the volatile composition of the fermented wine (Wei et al. 2022). The types, content, and ratio of esters, alcohols, and acids in wine play an important role in the organoleptic quality of rice wine, and the combined effect of the various aroma components influences the quality of rice wine (Yan et al. 2023). A total of 76 volatile flavor compounds were detected, including 15 alcohols, 33 esters, 8 acids, 5 aldehydes, 2 ketones, 3 volatile phenols, and 9 other volatile compounds in the 4 groups of rice wine. The specific types and content of their volatile flavor compounds in the four groups of rice wine are shown in Table S2.

**FIGURE 1:**
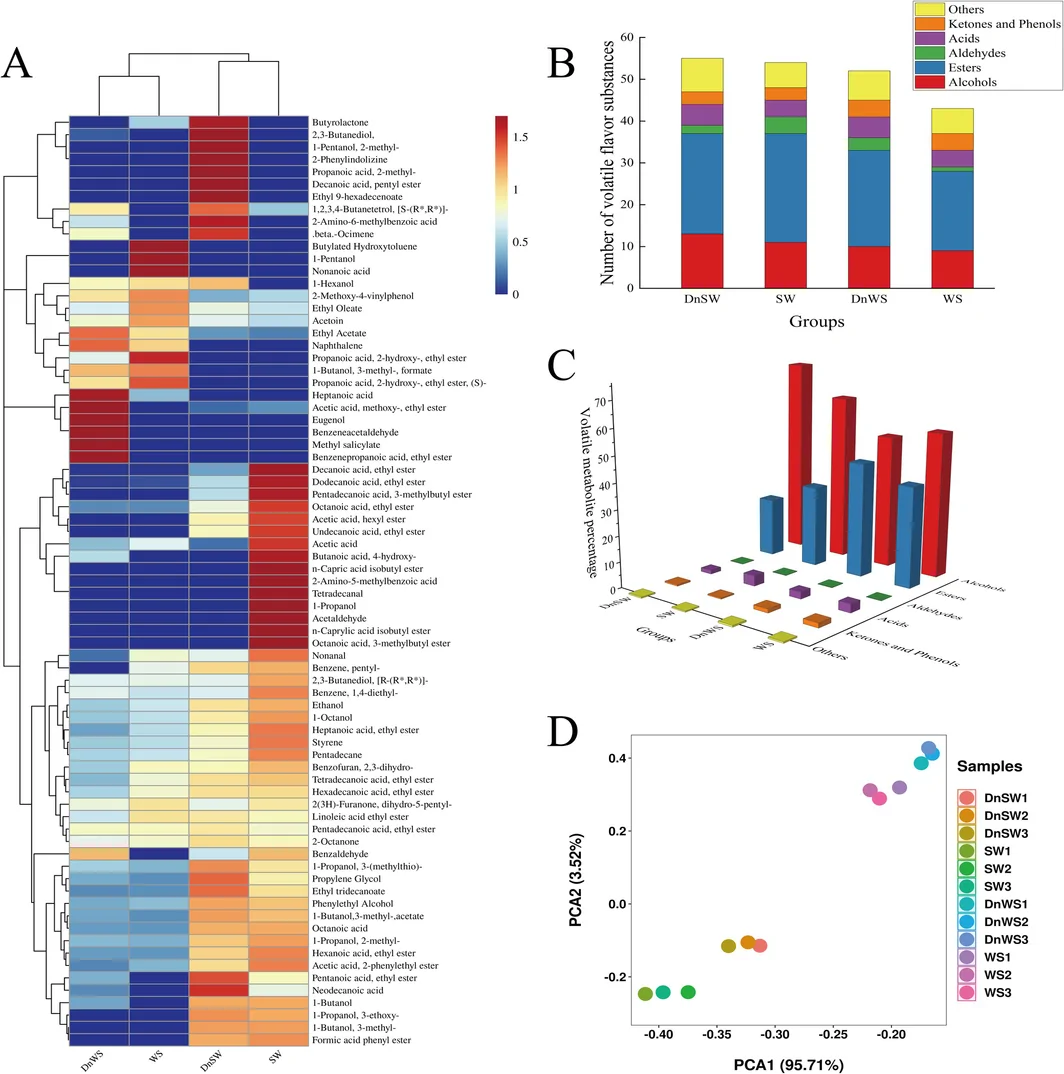
Hierarchical clustering heat map of volatile compounds in rice wine produced through different inoculation strategies (A), types of volatile flavor compounds in rice wine (B), percentage content of each flavor group (C), and principal component analysis of flavor compounds in rice wine (D).

Rice wine fermented by simultaneous inoculation of *Sc* and *Wa* had a higher content and richer variety of volatiles (Figure 1A,B). Rice wine fermented by sequential inoculation had a relatively low content and variety of volatile flavor compounds. The highest content of volatile flavor compounds was 32.49 mg/L in DnSW, and the most abundant types were detected, with a total of 55 types. There were significant changes in the volatile flavor compounds in rice wine in terms of type or content after the addition of *D. nobile*. Compared to SW, the content of volatile flavor compounds in DnSW increased 9.50 mg/L, without significant change in types. It is worth noting that compared to WS, the total content of flavor compounds in DnWS decreased by 2.56 mg/L, but the number of types increased by 9 compounds, suggesting that sequential inoculation fermentation is more conducive to the abundance of flavor compounds in *D. nobile* rice wine, which resulted in a more harmonized rice wine aroma. This may be due to the fact that *Wa* and its metabolites in sequentially inoculated fermented rice wine inhibited the later inoculated *Sc*, whereas there was less inhibition of *Sc* present in simultaneously inoculated fermented rice wine, resulting in a richer content of volatile flavor compounds. After the addition of *D. nobile*, there was a decrease in the content and types of alcohol in rice wine fermented by simultaneous inoculation, while the content of alcohols and esters in rice wine fermented by sequential inoculation was slightly reduced, and the types of esters were richer. The results showed that *D. nobile* would inhibit the formation of volatiles in mixed‐fungi fermented rice wine.

Rice wine fermented by simultaneous inoculation of *Sc* and *Wa* had a higher content and richer variety of volatiles (Figure 1A,B). Rice wine fermented by sequential inoculation had a relatively low content and variety of volatile flavor compounds. The highest content of volatile flavor compounds was 32.49 mg/L in DnSW, and the most abundant types were detected, with a total of 55 types. There were significant changes in the volatile flavor compounds in rice wine in terms of type or content after the addition of *D. nobile*. Compared to SW, the content of volatile flavor compounds in DnSW increased 9.50 mg/L, without significant change in types. It is worth noting that compared to WS, the total content of flavor compounds in DnWS decreased by 2.56 mg/L but the number of types increased by 9 compounds, suggesting that sequential inoculation fermentation is more conducive to the abundance of flavor compounds in *D. nobile* rice wine, which resulted in a more harmonized rice wine aroma. This may be due to the fact that *Wa* and its metabolites in sequentially inoculated fermented rice wine inhibited the later inoculated *Sc*, whereas there was less inhibition of *Sc* present in simultaneously inoculated fermented rice wine, resulting in a richer content of volatile flavor compounds. After the addition of *D. nobile*, there was a decrease in the content and types of alcohol in rice wine fermented by simultaneous inoculation, while the content of alcohols and esters in rice wine fermented by sequential inoculation was slightly reduced, and the types of esters were richer. The results showed that *D. nobile* would inhibit the formation of volatiles in mixed‐fungi fermented rice wine.

### 3.2 Analysis of Metabolites

#### 3.2.1 PCA Analysis of Metabolites

**FIGURE 2:**
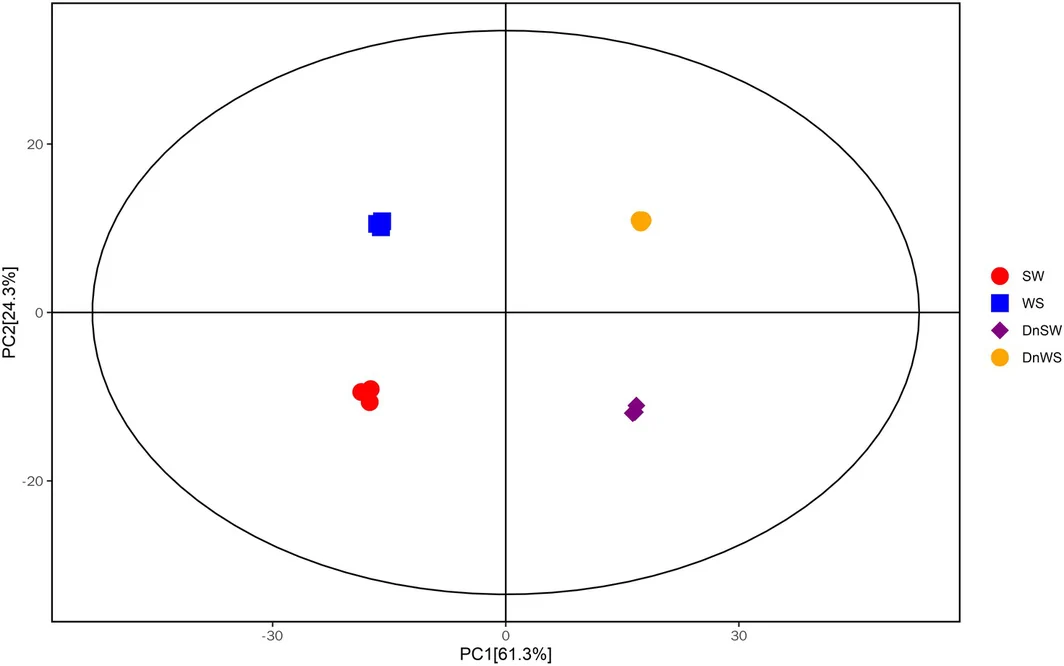
Principal component analysis of rice wine metabolites.

The metabolites identified in the four groups of rice wine were subjected to principal component analysis (Figure 2). Each group was well clustered, indicating that the samples had less within‐group variation and better reproducibility. The second principal components of WS were located in the positive semiaxis and SW in the negative semiaxis, whereas the first principal components were all located in the negative semiaxis, indicating that there was a difference in the metabolites of the rice wine from the simultaneous inoculated fermentation of *Sc* and *Wa* and from the sequential inoculated fermentation of the rice wine. The first principal components of rice wine with the addition of *D. nobile* were all located in the positive semiaxis, and those without the addition of *D. nobile* were all located in the negative semiaxis, indicating that *D. nobile* had a significant effect on the metabolites of the rice wine fermented with both simultaneous inoculation and sequential inoculation of *Sc* and *Wa*.

#### 3.2.2 Statistical Analysis of Metabolites Classification

The metabolites detected in each group of rice wine were further statistically analyzed and their relative content was reflected by the total peak area of the metabolites. The various metabolites of rice wine in the four groups were classified into 22 categories, including flavonoids, phenols, alkaloids, terpenoids, lignans, naphthalenes, astragalus, coumarins, steroids, sugars, amino acids, aromatic compounds, etc., and differed in type and content (Table 3). The relative percentages and detailed compound classification of major active metabolites are presented in Table S3. A total of 1268 metabolites were detected and identified in SW rice wine, 1279 in WS, 1327 in DnSW, and 1324 in DnWS, suggesting that the addition of *D. nobile* resulted in a greater abundance of metabolites in rice wine.

**TABLE 3:** Metabolite types and their peak areas in rice wine.

Name	SW		DnSW		WS		DnWS	
	Type	Peak area	Type	Peak area	Type	Peak area	Type	Peak area
Flavonoids	52	1.42 × 10^8^	65	2.45 × 10^8^	52	1.12 × 10^8^	64	1.72 × 10^8^
Phenols	13	1.45 × 10^8^	15	3.12 × 10^8^	15	1.14 × 10^8^	15	8.78 × 10^8^
Alkaloids	56	5.89 × 10^9^	58	1.18 × 10^9^	56	3.35 × 10^9^	56	8.48 × 10^9^
Terpenoids	87	3.22 × 10^8^	104	1.26 × 10^9^	89	3.53 × 10^8^	104	1.14 × 10^9^
Lignans	24	1.09 × 10^9^	25	1.09 × 10^9^	23	9.15 × 10^8^	23	5.14 × 10^9^
Coumarins	9	3.00 × 10^7^	9	6.04 × 10^7^	9	3.63 × 10^7^	9	5.90 × 10^7^
Steroids	33	1.13 × 10^8^	35	5.73 × 10^7^	34	2.76 × 10^8^	34	1.96 × 10^8^
Naphthalenes	11	5.34 × 10^7^	11	5.56 × 10^7^	11	6.38 × 10^7^	11	7.31 × 10^7^
Astragalus	9	5.51 × 10^7^	10	5.00 × 10^7^	9	5.35 × 10^7^	9	4.51 × 10^7^
Amino acids	250	7.62 × 10^9^	251	4.10 × 10^9^	252	1.06 × 10^10^	252	9.99 × 10^9^
Sugars	68	8.45 × 10^8^	70	1.12 × 10^9^	68	5.85 × 10^9^	69	7.98 × 10^9^
Ketones	7	1.91 × 10^8^	7	2.06 × 10^8^	7	6.69 × 10^7^	7	4.79 × 10^7^
Organic acids	27	9.31 × 10^8^	25	1.08 × 10^9^	27	1.56 × 10^9^	27	1.33 × 10^9^
Aromatic compounds	32	6.01 × 10^8^	32	2.85 × 10^8^	33	7.02 × 10^8^	32	1.34 × 10^9^
Glycerophospholipids	18	5.55 × 10^7^	18	1.20 × 10^8^	18	1.17 × 10^8^	18	9.26 × 10^7^
Nucleosides	16	9.33 × 10^8^	16	7.39 × 10^9^	16	5.49 × 10^9^	16	1.41 × 10^9^
Carbonyl compounds	15	1.07 × 10^9^	15	8.86 × 10^8^	15	1.18 × 10^9^	15	7.32 × 10^8^
Organic oxygen compounds	18	1.44 × 10^8^	18	1.19 × 10^8^	16	1.82 × 10^8^	18	7.51 × 10^8^
Organic heterocyclic compounds	112	1.01 × 10^9^	119	1.39 × 10^9^	114	1.61 × 10^9^	119	1.46 × 10^9^
Fatty acids	54	4.15 × 10^9^	54	3.36 × 10^9^	55	4.51 × 10^9^	55	2.95 × 10^9^
Fatty acyl	40	3.84 × 10^8^	42	1.29 × 10^9^	41	1.32 × 10^9^	42	6.58 × 10^8^
Others	317	3.84 × 10^10^	328	2.14 × 10^10^	319	1.22 × 10^10^	329	4.36 × 10^10^
Total	1268	6.42 × 10^10^	1327	4.71 × 10^10^	1279	5.07 × 10^10^	1324	8.86 × 10^10^

Compared to SW, DnSW rice wine was richer in flavonoids, phenols, terpenoids, and sugar metabolites, especially *Dendrobium* terpenoids, with a large increase in type and content, and this increase may come from *D. nobile* or the metabolic transformation of *D. nobile* by yeasts. It is noteworthy that there was no large increase in alkaloids, steroids, and amino acid metabolites in DnSW rice wine in terms of type, but there was a large decrease in the content of these compounds. Compared to WS, there was a large increase in the type and content of flavonoid and terpenoid metabolites in DnWS rice wine, and the changes in phenolic, alkaloid, coumarin, and naphthalene metabolites were only reflected in the elevated content.

There was no large difference in metabolite abundance between DnSW and DnWS, but large differed in content. There was no large difference in terpenes, coumarins, and other metabolites between the two groups of rice wine, but flavonoids, phenolics, alkaloids, lignans, steroids, amino acids, and sugar metabolites were higher in DnWS. Therefore, simultaneous inoculation promoted the metabolic transformation of flavonoids active compounds in *D. nobile* rice wine, and sequential inoculation promoted the metabolism of phenols, alkaloids, lignans, steroids, amino acids and other active compounds in *D. nobile* rice wine. Different inoculation strategies had no significant effect on the metabolism of terpenoids and coumarins active compounds in *D. nobile* rice wine. The high content of active compounds such as *Dendrobium* alkaloids, flavonoids and total phenols in DnWS rice wine fully demonstrated that sequential inoculation could induce active metabolites in *D. nobile* rice wine to actively participate in the fermentation of the rice wine and effectively enhance the nutritional value of the rice wine.

#### 3.2.3 Analysis of Metabolites Pathway Enrichment

Using the criteria of VIP > 1.0 and *p* < 0.05 (Student's *t*‐test), the four groups of rice wine were analyzed according to metabolic pathways, as well as metabolite enrichment and expression in the positive and negative ion modes of WS‐SW, DnWS‐DnSW, DnSW‐SW, and DnWS‐WS (Table 4). The metabolic pathways and metabolites of the four different fermented rice wine were larger different, and the effect of *D. nobile* on the fermentation and metabolism of rice wine was smaller than that of the different inoculation strategies of *Sc* and *Wa* on the fermentation and metabolism of rice wine. Because of the complexity of the metabolic pathways and metabolites involved in rice wine fermentation, specific differences need to be further analyzed.

**TABLE 4:** Overall expression trends of metabolic pathways and metabolites in rice wine.

Ion mode	WS‐SW		DnWS‐DnSW		DnSW‐SW		DnWS‐WS	
	ES+	ES−	ES+	ES−	ES+	ES−	ES+	ES−
Metabolites	561	524	565	534	490	441	527	489
Differential metabolites	311	308	281	242	145	128	228	167
Up‐Metabolites	212	232	193	136	114	122	158	89
Up‐Metabolic pathway	54	55	52	51	40	37	59	39
Down‐Metabolites	97	76	81	106	31	6	70	78
Down‐Metabolic pathway	37	31	27	41	15	5	34	31

*Note:* ES+ indicates positive ion mode and ES− indicates negative ion mode.

#### 3.2.4 Analysis of Metabolic Differences Between SW and WS

**FIGURE 3:**
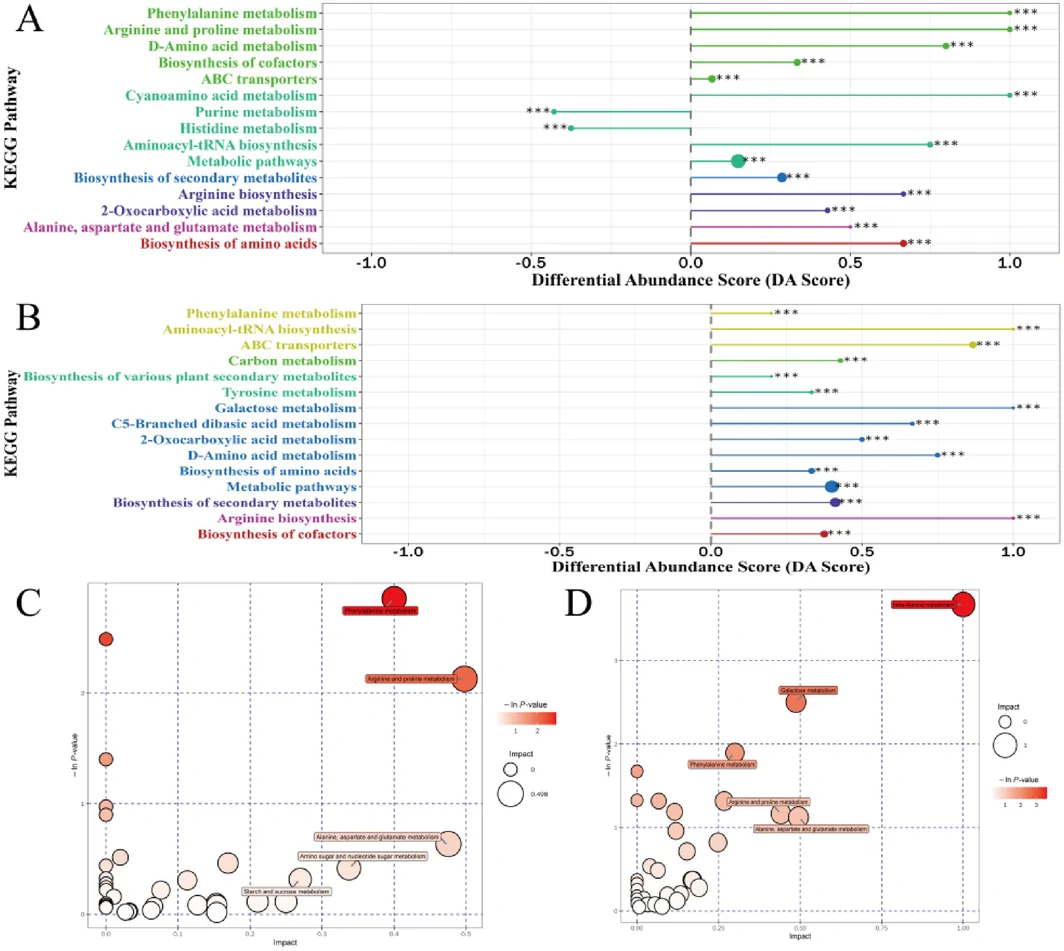
Differential score plots of WS‐SW rice wine metabolic pathways: positive ion mode (A), positive ion mode (B); WS‐SW rice wine pathway analysis plots: positive ion mode (C), negative ion mode (D). Asterisks indicate significant differences (***P < 0.001).

The overall expression of metabolic pathways in WS‐SW in the positive ion mode showed mostly an up‐regulation trend. More metabolites were obtained through these metabolic pathways in WS rice wine than in SW (Figure 3A,B). Among them, phenylalanine metabolism, arginine and proline metabolism, D‐amino acid metabolism and biosynthesis of cofactors belong to the amino acid metabolic pathway. Notably, two metabolic pathways showed a trend of down‐regulation of the expression of the annotated differential metabolites. Histidine metabolism was enriched for differential metabolites such as L‐glutamic acid, histamine, L‐histidine, imidazole‐1‐acetic acid, L‐histidinol, methylisoimidazoleacetic acid, and so forth, and purine metabolism was enriched for differential metabolites such as guanine, 2′‐deoxyadenosine, guanine nucleoside, glycine, and so on. It has been shown that histidine can effectively prevent the inactivation of AMP deaminase and can act as a physiological “antioxidant” in yeast cells (Murakami et al. 1997). Nucleotide metabolism facilitates the synthesis and expression of various key genes and enzymes in yeast growth and fermentation. It indicates that SW rice wine produces more differential metabolites through these two metabolic pathways. The activities such as growth, metabolism and fermentation of yeast were more active.

The overall expression of all metabolic pathways in WS‐SW in negative ion mode showed an up‐regulation trend. Among them, biosynthesis of various plant secondary metabolites and tyrosine metabolism belonged to the sugar metabolism pathway. Differential metabolites enriched in the biosynthesis of various plant secondary metabolites were o‐coumaric acid, L‐phenylalanine, and trans‐cinnamic acid, and tyrosine metabolism was enriched in maleic acid, norepinephrine, p‐hydroxyphenylethylamine, and DL‐dopa, etc. Phenylalanine metabolism, aminoacyl‐tRNA biosynthesis, and so forth, belong to the amino acid metabolic pathway. Differential metabolites enriched in phenylalanine metabolism were o‐coumaric acid, L‐phenylalanine, trans‐cinnamic acid, and phenylacetic acid, and aminoacyl‐tRNA biosynthesis was enriched in L‐arginine, L‐glutamic acid, L‐phenylalanine, and L‐serine. O‐coumaric acid and trans‐cinnamic acid are very important active ingredients that ultimately confer a certain biological activity to rice wine. Differential metabolites such as L‐arginine, L‐glutamic acid, L‐phenylalanine, and L‐serine are ultimately biologically transformed by yeast to form important aromatic compounds, active ingredients, and so on in rice wine. In rice wine fermentation, yeast converts free amino acids in rice wine into higher alcohols through amino acid metabolism or glucose into higher alcohols through sugar metabolism (El‐Dalatony et al. 2019). The differences in the higher alcohols and sugar content of the two groups of rice wine may arise from these metabolic pathways. Previous studies have demonstrated that rice wine fermented with simultaneous inoculation of *Sc* and *Wa* produces more higher alcohols, whereas rice wine fermented with sequential inoculation has a lower conversion rate of sugar utilization, resulting in the accumulation of large amounts of sugar in rice wine (Yao et al. 2023). SW contains more higher alcohols and less total sugars, reducing sugars, and polysaccharides than WS, mainly because the yeast in SW can efficiently utilize the sugars and amino acids to form higher alcohols.

The metabolic pathways with large differences in the WS‐SW positive ion patterns belonged to the amino acid metabolic pathway (Figure 3C). The largest discrepancy was in phenylalanine metabolism, with differential metabolites annotated as phenylacetaldehyde, 2‐Phenylacetamide, L‐Phenylalanine, and so forth. Next was arginine and proline metabolism, annotated to differential metabolites such as L‐arginine, L‐glutamic acid, and L‐proline. In the negative ion pattern (Figure 3D), beta‐Alanine metabolism was the most differential metabolic pathway, and the differential metabolite annotated was pantothenic acid. This was followed by galactose metabolism, annotated to cotton seed sugar.

All of these are amino acid metabolism except galactose metabolism, which is sugar metabolism. In the Galactose metabolism pathway, cotton seed sugar produces sucrose in the presence of galactosidase, and then sucrose is converted to glucose and fructose in the presence of convertases (Chen et al. 2022). This also resulted in a significantly higher sugar content in WS rice wine than in SW rice wine. Amino acids contribute to the various flavors of fermented rice wine, including freshness, sweetness, bitterness and astringency characteristics, and are important nutritional and flavor precursors in rice wine, providing a source of nitrogen for the growth of microorganisms during fermentation (Liang et al. 2020). Yeast can produce heterologous compounds such as aromatic compounds, flavonoids, and phenols by utilizing glucose through the amino acid pathway (Gottardi et al. 2017). Therefore, WS and SW mainly differ in flavor compounds, flavonoids produced through these differential metabolic pathways. Although previous studies have observed differences in flavor and active components between the two inoculation strategies, the present study further demonstrates that when fermenting rice wine with *Sc* and *Wa*, sequential inoculation is more conducive to the accumulation of functional substances (e.g., flavonoids and total phenols) compared to simultaneous inoculation, whereas simultaneous inoculation favors a higher total content of volatile flavor compounds and enrichment of alcohols.

#### 3.2.5 Metabolic Effects of *D. nobile* on *Sc* and *Wa* Fermented Rice Wine

**FIGURE 4:**
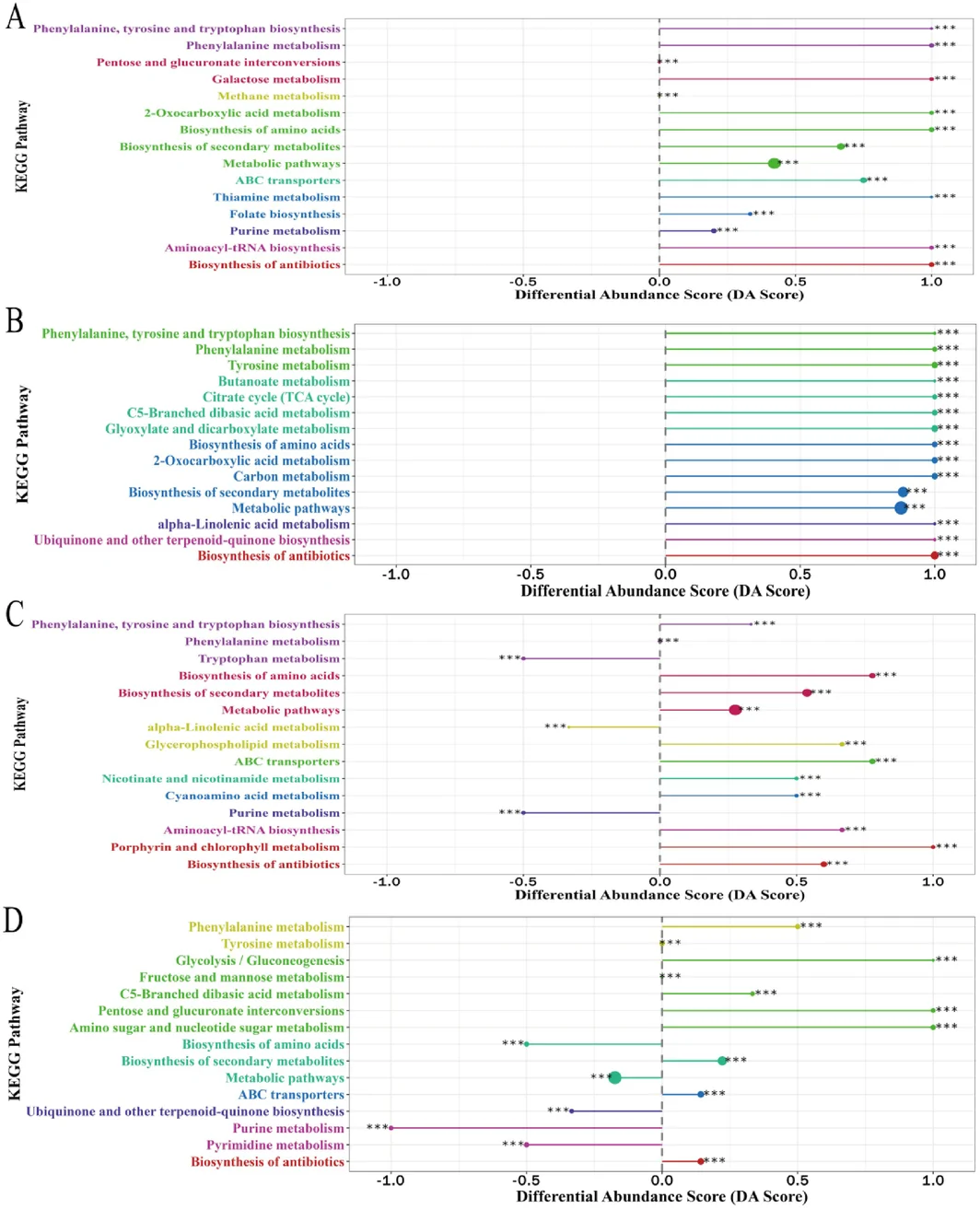
Differential score of metabolic pathways of rice wine, DnSW‐SW positive ion mode (A), DnSW‐SW negative ion mode (B), DnWS‐WS positive ion mode (C), and DnWS‐WS negative ion mode (D). Asterisks indicate significant differences (**P < 0.01, ***P < 0.001).

The effect of *D. nobile* on the metabolism of the two yeast strains during fermentation with different inoculation strategies was analyzed by a two‐by‐two comparison strategy of DnSW‐SW and DnWS‐WS (Figure 4). The overall expression of all metabolic pathways tended to be up‐regulated in the DnSW‐SW positive and negative ion patterns, suggesting that *D. nobile* had a significant effect on the metabolic pathways of rice wine fermented with simultaneous inoculation of *Sc* and *Wa*. The metabolites enriched through these metabolic pathways were more in DnSW and less in SW, and therefore, the metabolite differences in rice wine after addition of *D. nobile* were significant. Five metabolic pathways belonged to sugar metabolism: pentose and glucuronate interconversions were enriched in 2 differential metabolites, glycerol and dehydroisostanone 3‐glucuronide; galactose metabolism was enriched in glycerol; and butanoate metabolism was enriched in maleic acid; C5‐Branched dibasic acid metabolism was enriched to trans‐aconitic acid, methyl fumaric acid, citraconic acid; glyoxylate and dicarboxylate metabolism was enriched to methyl fumaric acid. It has been shown that yeast stressed in a high sugar environment ensures yeast activity by preferentially increasing the intensity of alginate, glycerol and glycolytic metabolism, attenuating tricarboxylic acid metabolism, and first attenuating and then increasing pentose phosphate metabolism (Xie et al. 2022). Through the tricarboxylic acid cycle (TCA cycle), organic acids such as citric acid, L‐malic acid and succinic acid are formed, which play an important role in the formation of wine flavor (Jiang et al. 2020). This could explain the greater abundance of volatile flavor compounds in SW rice wine in the previous result. There were also three amino acid metabolic pathways whose overall expression was up‐regulated: phenylalanine, tyrosine and tryptophan biosynthesis enriched to L‐phenylalanine, L‐tyrosine; phenylalanine metabolism enriched to L‐phenylalanine, phenylglyoxal, L‐tyrosine, 2‐phenylacetamide, trans‐cinnamic acid; tyrosine metabolism enriched to L‐tyrosine, maleic acid, norepinephrine, acetamide, trans‐cinnamic acid; tyrosine metabolism enriched to L‐tyrosine, maleic acid, norepinephrine. L‐Phenylalanine and L‐tyrosine are precursors of phenolic compounds (e.g., trans‐cinnamic acid) and volatile aroma compounds (e.g., phenylethanol), which enhance the antioxidant capacity and flavor of rice wine. This metabolic reprogramming explains why DnSW had the highest total content of volatile flavor compounds (32.49 mg/L) and the most abundant variety (55 types) (Table S2), and also supports the observation that after *D. nobile* addition, the contents of alcohols and esters were higher in simultaneously inoculated rice wine. Therefore, under simultaneous inoculation conditions, *D. nobile* synergistically enhances the flavor intensity and complexity of rice wine by activating multiple metabolic pathways.

The DnWS‐WS positive–negative ion pattern had metabolic pathways with an overall up‐regulated expression trend, as well as a down‐regulated expression trend. The metabolic effects of *D. nobile* on *Sc* and *Wa* sequentially inoculated rice wine were more complex, with both promotional and some inhibitory effects. The overall expression of most metabolic pathways in the positive ion mode DnWS‐WS tended to be up‐regulated. The metabolic pathways whose overall expression tends to be down‐regulated include amino acid metabolism, lipid metabolism, and nucleotide metabolism. Tryptophan metabolism enriched to differential metabolites such as indole, yellow uric acid, and L‐kynurenine. The alpha‐Linolenic acid metabolism enriched to cis‐6, 9, 12, 15‐octadecatetraenoic acid and phosphatidylserine. Purine metabolism was enriched in guanine, 2′‐deoxyadenosine, and guanine nucleoside. DnWS rice wine fermentation has fewer metabolites converted and produced through them than WS. The up‐regulated and down‐regulated metabolic pathways in the negative ion model were almost equal. The metabolic pathways whose overall expression tended to be down‐regulated were purine metabolism and pyrimidine metabolism, both of which belong to nucleotide metabolism; the former was enriched in 5‐amino‐1H‐imidazole‐4‐amide, 2′‐deoxyadenosine, and hypoxanthine; and the latter was enriched in uracil, spongouridine, and pseudouridine. The metabolic pathways such as Glycolysis/Gluconeogenesis, pentose and glucuronate interconversions, and amino sugar and nucleotide sugar metabolism, whose overall expression was more inclined to be up‐regulated, belonged to sugar metabolism; the first metabolic pathway was enriched for the differential metabolite D‐glucose; the second was enriched to D‐xylulose and D‐xylose; and the third was enriched to D‐glucose and D‐xylose. More metabolites were converted and produced through these sugar metabolism pathways in DnWS rice wine fermentation than in WS. Our previous results have shown that DnWS rice wine contains more polysaccharides, total sugars, and reducing sugars, which may be due to the fact that *D. nobile* can drive sequential inoculation fermentation to degrade large fractions of compounds through sugar metabolism, thus promoting the transformative effect of microorganisms on the utilization of small molecules. The down‐regulation of nucleotide metabolism in DnWS may indicate reduced nucleic acid turnover, possibly due to slower yeast growth or metabolic adaptation to *D. nobile*. The simultaneous occurrence of up‐regulated and down‐regulated pathways reflects the complex, bidirectional regulatory effect of *D. nobile* on sequential fermentation. These metabolic adjustments collectively underpin the high sugar content and unique flavor profile of DnWS rice wine.

**FIGURE 5:**
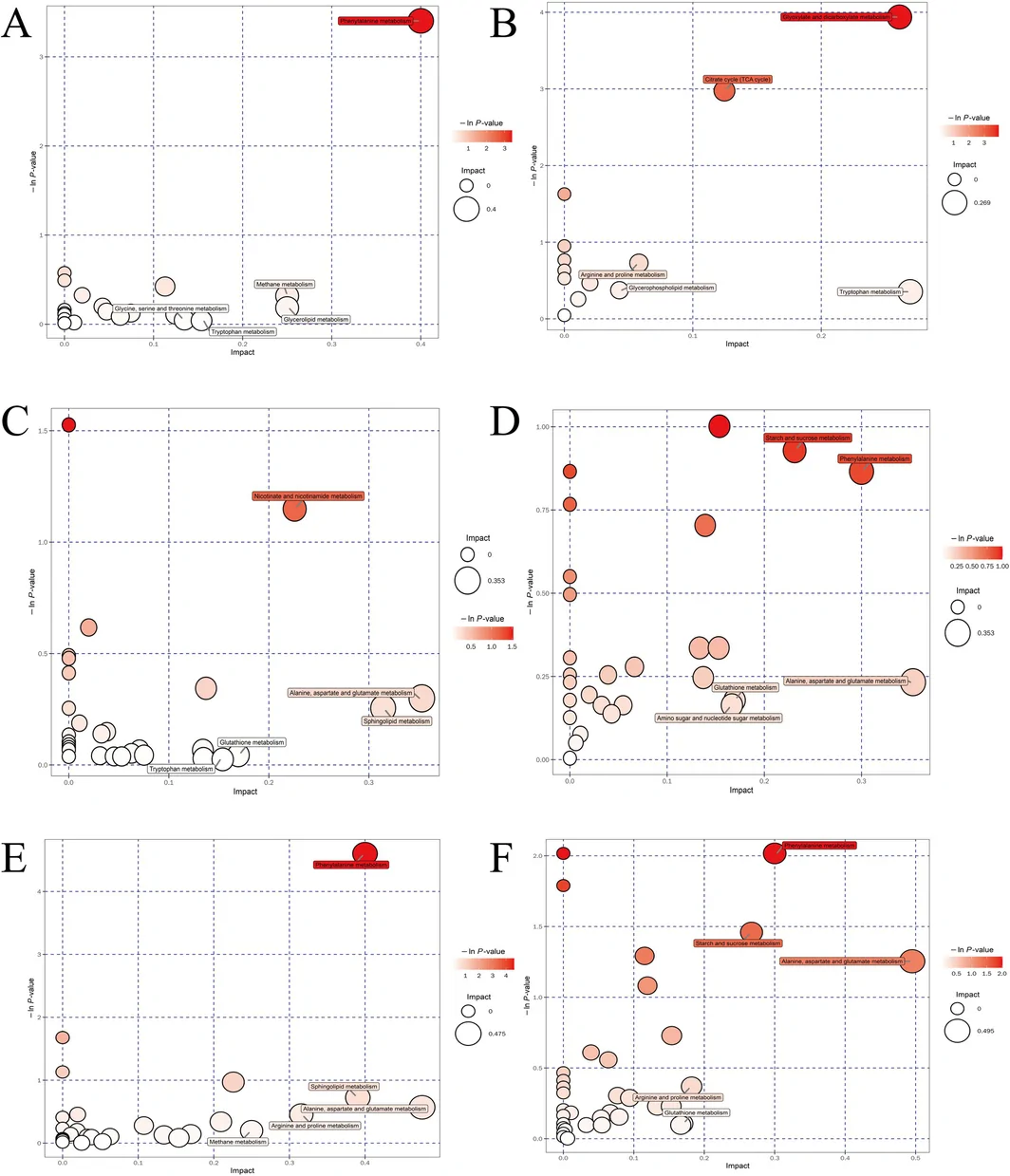
Rice wine pathway analysis diagram: DnSW‐SW positive ion mode (A), DnSW‐SW negative ion mode (B), DnWS‐WS positive ion mode (C), DnWS‐WS negative ion mode (D), DnWS‐DnSW positive ion mode (E), DnWS‐DnSW negative ion mode (F).

The addition of *D. nobile* had a large effect on the metabolites of *Sc* and *Wa* fermented rice wine, with a high number of differential metabolites, and the metabolic pathways involved were numerous and complex. Among the metabolic pathways with large differences were shown in Figure 5A,B. The metabolic pathway with the most significant difference in DnSW‐SW positive ion patterns was phenylalanine metabolism, and the differential metabolites hitting this pathway were phenylacetaldehyde, 2‐phenylacetamide, and L‐phenylalanine. The most significant difference in negative ion patterns was glyoxylate and dicarboxylate metabolism, and the differential metabolites hitting this pathway were cis‐aconitic acid, isocitric acid, and succinic acid. Studies have shown that yeast can produce the aromatic compound phenylethanol with a rose flavor through the phenylalanine metabolic pathway (Wittmann et al. 2002). This is in agreement with the previous result that the phenylethanol content of DnSW rice wine was significantly increased by the addition of *D. nobile*, which was 1.68 times higher than that of SW. The metabolic pathway with the most significant DnWS‐WS positive ion pattern differences was mainly Nicotinate and nicotinamide metabolism (Figure 5C,D), and the differential metabolite that hit it was mainly nicotinamide adenine dinucleotide. The most significant differences in negative ion patterns were in Starch and sucrose metabolism and Phenylalanine metabolism, with both pathways being hit by the differential metabolite D‐glucose. Glucose can be produced through starch and sucrose metabolism in rice wine fermentation (Jiang et al. 2020). Thus the total sugar content in DnWS rice wine was higher than in WS, and this increased sugar was mainly produced by sugar metabolism.

DnWS‐DnSW positive and negative ion patterns, the metabolic pathway that differed most between the two groups of rice wine was Phenylalanine metabolism (Figure 5E,F), and the differential compounds hitting this metabolic pathway were phenylacetaldehyde, 2‐Phenylacetamide, L‐Phenylalanine, phenylacetic acid, and phenylacetylglycine. In addition, metabolic pathways with large differences in negative ion patterns were starch and sucrose metabolism (the differential metabolite was alginate) and alanine, aspartate and glutamate metabolism (the differential metabolite was L‐glutamate). Among them, phenylalanine metabolism and arginine and proline metabolism were the differential metabolic pathways common to both unadded *D. nobile* and *D. nobile*‐added fermented rice wine, suggesting that these two pathways mainly originated from the differences in the different fermentation sequences of the two yeast strains. The addition of *D. nobile* affects the production of differential metabolites through these two metabolic pathways, promoting the production of differential metabolites in phenylalanine metabolism and inhibiting the production of differential metabolites in arginine and proline metabolism.

Phenylalanine metabolism was the most divergent pathway between DnWS and DnSW, and it also appeared as a common differential pathway in the comparison with and without *D. nobile* addition (SW vs. WS), indicating that phenylalanine metabolism is highly sensitive to both inoculation strategy and *D. nobile* addition. The promotion of phenylalanine metabolism by *D. nobile* in DnWS (compared to DnSW) is consistent with the higher levels of phenolic compounds and phenylethanol in DnWS rice wine, contributing to enhanced antioxidant activity and floral/rose‐like aroma. Conversely, the suppression of arginine and proline metabolism by *D. nobile* may reduce the production of certain higher alcohols or polyamines, potentially affecting mouthfeel and flavor complexity. Meanwhile, the up‐regulation of starch and sucrose metabolism in DnWS further supports the higher sugar content in DnWS rice wine. Collectively, these metabolic differences explain why, compared to DnSW, DnWS exhibits both stronger functional components (via the phenylalanine pathway) and a unique sugar accumulation pattern.

## 4 Conclusion

The different inoculation strategies of *Sc* and *Wa* and the addition of *D. nobile* had a greater effect on the physicochemical indexes and active compounds of rice wine. The content of all the active compounds in rice wine were notably enhanced by the addition of *D. nobile*. Sequential inoculation of *Sc* and *Wa* promoted the formation and release of active compounds in the fermentation of *D. nobile* rice wine more than simultaneous inoculation. Simultaneously inoculated fermented rice wine were higher in volatile flavor compounds and richer in variety, with a large proportion of alcohols, while sequential inoculated fermentation increased the proportion of esters in rice wine. *D. nobile* inhibits the increase in the content of flavor compounds in rice wine, but enhances the abundance of flavor compounds. The metabolic pathways and their metabolites of rice wine were significantly different as a result of different inoculation strategies of *Sc* and *Wa* and the addition of *D. nobile*. The widely varying metabolic pathway phenylalanine metabolism in rice wine is mainly likely to be due to *D. nobile* and the different inoculation strategies. Differential metabolic pathways of arginine and proline metabolism, galactose metabolism, and starch and sucrose metabolism result mainly from different inoculation strategies. Glyoxylate and dicarboxylate metabolism, nicotinate and nicotinamide metabolism metabolic pathway differences were mainly caused by the addition of *D. nobile*.

## Author Contributions

**Hongling Gan:** writing – review and editing, writing – original draft. **Guichun Huang:** software. **Lin Zhang:** methodology. **Lanyan Cen:** validation. **Yifeng Dai:** supervision. **Shuyi Qiu:** project administration. **Xiaodan Wang:** supervision. **Chaoyang Wei:** funding acquisition, supervision.

## Funding

This work was supported by the National Natural Science Foundation of China (No. 32160566) and the National Natural Science Foundation of China (No. 32460591), Guizhou Key Laboratory of New Quality Processing and Storage of Ecological Specialty Food (No. ZSYS[2025]023), the Guizhou High‐level Innovative Talents Project (Guizhou Science and Technology Cooperation Platform Talented‐GCC[2023]013), Industry‐University‐Research Cooperation Project (K22‐0515‐001), Construction of scientific and technological innovation talents team for mining and utilizing characteristic microbial resources of fermentation industry in Guizhou Province (Guizhou Science and Technology Cooperation Platform Talented CXTD[2023]029), Laboratory Opening Project of Guizhou University (SYSKF2023‐072).

## Conflicts of Interest

The authors declare no conflicts of interest.

## Supporting information


**Figure S1:** CO_2_ weight loss diagram of rice wine fermented by mixed fungi with different ratios of *Sc* and *Wa* (A), Variation of CO_2_ weight loss of rice wine with different amounts of *D. nobile*. (B), Changes in CO_2_ weight loss of rice wine fermented with different inoculation sequences of *Sc* and *W*a mixed fungi (C) Changes in CO_2_ weight loss of rice wine (D).
**Figure S2:** Physicochemical indexes of rice wine fermented with different ratios of Sc and Wa, (A) total sugar content, (B) polysaccharide content, (C) reducing sugar content, (D) total flavonoid content, (E) total phenols content.
**Figure S3:** Physicochemical indexes of rice wine with different additions of Dendrobium nobile Lindl., (A) total sugar content, (B) polysaccharide content, (C) total flavonoid content, (D) total phenols content.
**Figure S4:** Physicochemical indexes of rice wine fermented in different inoculation sequences of Sc and Wa, (A) total sugar content, (B) reducing sugar content, (C) polysaccharide content, (D) total phenols content, (E) total flavonoid content.
**Table S1:** Sensory evaluation of rice wine.
**Table S2:** Volatile substances and their contents (μg/L) in rice wine.
**Table S3:** Relative percentage of major active metabolites in rice wine.

## Data Availability

All data supporting the findings of this study are available within the Supporting Information of this article.

## References

[fsn372321-bib-0001] Albalasmeh, A. A. , A. A. Berhe , and T. A. Ghezzehei . 2013. “A New Method for Rapid Determination of Carbohydrate and Total Carbon Concentrations Using UV Spectrophotometry.” Carbohydrate Polymers 97, no. 2: 253–261. 10.1016/j.carbpol.2013.04.072. 23911443

[fsn372321-bib-0002] Canonico, L. , E. Galli , A. Agarbati , F. Comitini , and M. Ciani . 2021. “ *Starmerella bombicola* and *Saccharomyces cerevisiae* in Wine Sequential Fermentation in Aeration Condition: Evaluation of Ethanol Reduction and Analytical Profile.” Food 10, no. 5: 1047. 10.3390/foods10051047. PMC8151965 34064665

[fsn372321-bib-0003] Cen, L. , X. Shi , L. Zhang , et al. 2023. “Effects of Dendrobium Officinale on the Quality of Rice Wine Fermented Separately by *Saccharomyces cerevisiae* and *Wickerhamomyces anomalus*: Physicochemical Indices, Volatile Compounds and Nonvolatile Metabolites.” Fermentation 9, no. 7: 627. 10.3390/fermentation9070627.

[fsn372321-bib-0004] Chen, L. H. , D. N. Li , and Y. Z. Rong . 2022. “Fermentation Mechanism of Ginkgo Rice Wine Using an Ultra‐High‐Performance Liquid Chromatography‐Quadrupole/Time‐Of‐Flight Mass Spectrometry Based Metabolomics Method.” Journal of Food Composition and Analysis 105: 104230. 10.1016/j.jfca.2021.104230.

[fsn372321-bib-0005] Comitini, F. , A. Agarbati , L. Canonico , and M. Ciani . 2021. “Yeast Interactions and Molecular Mechanisms in Wine Fermentation: A Comprehensive Review.” International Journal of Molecular Sciences 22, no. 14: 7754. 10.3390/ijms22147754. 34299371 PMC8307806

[fsn372321-bib-0006] El‐Dalatony, M. M. , S. Saha , S. P. Govindwar , R. A. Abou‐Shanab , and B.‐H. Jeon . 2019. “Biological Conversion of Amino Acids to Higher Alcohols.” Trends in Biotechnology 37, no. 8: 855–869. 10.1016/j.tibtech.2019.01.011. 30871798

[fsn372321-bib-0007] Escudero‐Lopez, B. , I. Cerrillo , G. Herrero‐Martin , et al. 2013. “Fermented Orange Juice: Source of Higher Carotenoid and Flavanone Contents.” Journal of Agricultural and Food Chemistry 61, no. 37: 8773–8782. 10.1021/jf401240p. 24004007

[fsn372321-bib-0008] Fang, R. , W. Zhou , and Q. Chen . 2019. “Ethyl Carbamate Regulation and Genomic Expression of *Saccharomyces cerevisiae* During Mixed‐Culture Yellow Rice Wine Fermentation With *Lactobacillus* sp.” Food Chemistry 292: 90–97. 10.1016/j.foodchem.2019.04.014. 31054697

[fsn372321-bib-0009] Gottardi, M. , M. Reifenrath , E. Boles , and J. Tripp . 2017. “Pathway Engineering for the Production of Heterologous Aromatic Chemicals and Their Derivatives in *Saccharomyces cerevisiae* : Bioconversion From Glucose.” FEMS Yeast Research 17, no. 4: fox035. 10.1093/femsyr/fox035. 28582489

[fsn372321-bib-0010] Hu, K. , G. J. Jin , W. C. Mei , T. Li , and Y. S. Tao . 2018. “Increase of Medium‐Chain Fatty Acid Ethyl Ester Content in Mixed *H. uvarum* / *S. cerevisiae* Fermentation Leads to Wine Fruity Aroma Enhancement.” Food Chemistry 239: 495–501. 10.1016/j.foodchem.2017.06.151. 28873596

[fsn372321-bib-0011] Huang, Y. C. , M. Q. Zhong , S. Y. Mu , et al. 2023. “Assessment of the Contributions of *Saccharomyces cerevisiae* , *Hansenula* sp. and *Pichia kudriavzevii* to Volatile Organic Compounds and Sensory Characteristics of Waxy Rice Wine.” European Food Research and Technology 249, no. 3: 685–697. 10.1007/s00217-022-04165-x.

[fsn372321-bib-0012] Jiang, L. , Y. Mu , S. Wei , Y. Mu , and C. Zhao . 2020. “Study on the Dynamic Changes and Formation Pathways of Metabolites During the Fermentation of Black Waxy Rice Wine.” Food Science & Nutrition 8, no. 5: 2288–2298. 10.1002/fsn3.1507. 32405386 PMC7215209

[fsn372321-bib-0013] Li, A. N. , L. Xiong , Z. J. Huang , et al. 2025. “Enhancing the Quality and Flavor of Pomelo Wine Through Sequential Fermentation With Schizosaccharomyces Pombe and *Saccharomyces cerevisiae* : A Non‐Targeted Metabolomic Analysis.” Food Bioscience 63: 105585. 10.1016/j.fbio.2024.105585.

[fsn372321-bib-0014] Li, D.‐D. , C.‐Q. Zheng , F. Zhang , and J.‐S. Shi . 2022. “Potential Neuroprotection by Dendrobium Nobile Lindl Alkaloid in Alzheimer's Disease Models.” Neural Regeneration Research 17, no. 5: 972–977. 10.4103/1673-5374.324824. 34558510 PMC8552836

[fsn372321-bib-0015] Li, K.‐L. , Y.‐M. Liang , Z. Chen , et al. 2024. “Genome‐Wide Identification of the Alkaloid Synthesis Gene Family CYP450, Gives New Insights Into Alkaloid Resource Utilization in Medicinal Dendrobium.” International Journal of Biological Macromolecules 259: 129229. 10.1016/j.ijbiomac.2024.129229. 38211913

[fsn372321-bib-0016] Liang, Z.‐C. , X.‐Z. Lin , Z.‐G. He , H. Su , W.‐X. Li , and Q.‐Q. Guo . 2020. “Comparison of Microbial Communities and Amino Acid Metabolites in Different Traditional Fermentation Starters Used During the Fermentation of Hong Qu Glutinous Rice Wine.” Food Research International 136: 109493. 10.1016/j.foodres.2020.109329. 32846528

[fsn372321-bib-0017] Liu, S. X. , O. Laaksonen , M. Kortesniemi , M. Kalpio , and B. R. Yang . 2018. “Chemical Composition of Bilberry Wine Fermented With Non‐Saccharomyces Yeasts (*Torulaspora delbrueckii* and *Schizosaccharomyces pombe*) and *Saccharomyces cerevisiae* in Pure, Sequential and Mixed Fermentations.” Food Chemistry 266: 262–274. 10.1016/j.foodchem.2018.06.003. 30381185

[fsn372321-bib-0018] Liu, S. X. , O. Laaksonen , and B. R. Yang . 2019. “Volatile Composition of Bilberry Wines Fermented With Non‐Saccharomyces and Saccharomyces Yeasts in Pure, Sequential and Simultaneous Inoculations.” Food Microbiology 80: 25–39. 10.1016/j.fm.2018.12.015. 30704594

[fsn372321-bib-0019] Liu, W. , R. Ji , A. Aimaier , et al. 2023. “Adjustment of Impact Phenolic Compounds, Antioxidant Activity and Aroma Profile in Cabernet Sauvignon Wine by Mixed Fermentation of Pichia Kudriavzevii and *Saccharomyces cerevisiae* .” Food Chemistry: X 18: 100685. 10.1016/j.fochx.2023.100685. 37131849 PMC10149247

[fsn372321-bib-0020] Lou, H. , X. Han , B. Fan , et al. 2023. “The Effect of Incorporating Lingonberry ( *Vaccinium vitis‐idaea* L.) on the Physicochemical, Nutrient, and Sensorial Properties of Chinese Sweet Rice Wine.” Journal of Food Measurement and Characterization 17, no. 3: 2932–2943. 10.1007/s11694-023-01834-7.

[fsn372321-bib-0021] Luan, Y. , B. Q. Zhang , C. Q. Duan , and G. L. Yan . 2018. “Effects of Different Pre‐Fermentation Cold Maceration Time on Aroma Compounds of Co‐Fermentation With *Hanseniaspora opuntiae* or *Pichia kudriavzevii* .” LWT‐ Food Science and Technology 92: 177–186. 10.1016/j.lwt.2018.02.004.

[fsn372321-bib-0022] Mercado‐Pacheco, J. , Y. Julio‐Altamiranda , E. Sánchez‐Tuirán , Á. D. González‐Delgado , and K. A. Ojeda . 2020. “Variables Affecting Delignification of Corn Wastes Using Urea for Total Reducing Sugars Production.” ACS Omega 5, no. 21: 12196–12201. 10.1021/acsomega.0c00645. 32548402 PMC7271402

[fsn372321-bib-0023] Murakami, K. , Y. Onoda , J. Kimura , and M. Yoshino . 1997. “Protection by Histidine Against Oxidative Inactivation of AMP Deaminase in Yeast.” Biochemistry and Molecular Biology International 42, no. 5: 1063–1069. 10.1080/15216549700203521. 9285075

[fsn372321-bib-0024] Ren, N. , W. W. Gong , Y. C. Zhao , D. G. Zhao , and Y. W. Xu . 2023. “Innovation in Sweet Rice Wine With High Antioxidant Activity: *Eucommia ulmoides* Leaf Sweet Rice Wine.” Frontiers in Nutrition 9: 1108843. 10.3389/fnut.2022.1108843. 36704789 PMC9871602

[fsn372321-bib-0025] Tounsi, M. S. , W. A. Wannes , I. Ouerghemmi , et al. 2011. “Juice Components and Antioxidant Capacity of Four Tunisian Citrus Varieties.” Journal of the Science of Food and Agriculture 91, no. 1: 142–151. 10.1002/jsfa.4164. 20862741

[fsn372321-bib-0026] Wang, S. , H. Wu , P. Geng , et al. 2016. “Pharmacokinetic Study of Dendrobine in Rat Plasma by Ultra‐Performance Liquid Chromatography Tandem Mass Spectrometry.” Biomedical Chromatography 30, no. 7: 1145–1149. 10.1002/bmc.3641. 26525040

[fsn372321-bib-0027] Wei, R. , Y. Ding , N. Chen , et al. 2022. “Diversity and Dynamics of Microbial Communities During Spontaneous Fermentation of Cabernet Sauvignon ( *Vitis vinifera* L.) From Different Regions of China and Their Relationship With the Volatile Components in the Wine.” Food Research International 156: 111372. 10.1016/j.foodres.2022.111372. 35650985

[fsn372321-bib-0028] Wittmann, C. , M. Hans , and W. Bluemke . 2002. “Metabolic Physiology of Aroma‐Producing *Kluyveromyces marxianus* .” Yeast 19, no. 15: 1351–1363. 10.1002/yea.920. 12402244

[fsn372321-bib-0029] Xie, D. , Y. Sun , and Y. Lei . 2022. “Effect of Glucose Levels on Carbon Flow Rate, Antioxidant Status, and Enzyme Activity of Yeast During Fermentation.” Journal of the Science of Food and Agriculture 102, no. 12: 5333–5347. 10.1002/jsfa.11887. 35318660

[fsn372321-bib-0030] Xu, A. , Y. Xiao , Z. He , et al. 2022. “Use of Non‐Saccharomyces Yeast co‐Fermentation With *Saccharomyces cerevisiae* to Improve the Polyphenol and Volatile Aroma Compound Contents in Nanfeng Tangerine Wines.” Journal of Fungi 8, no. 2: 128. 10.3390/jof8020128. 35205881 PMC8875693

[fsn372321-bib-0031] Yan, Z. , L. V. Linling , H. Luo , and Z. Jin . 2023. “Effects of Different Koji on Aroma Components of Rice Wine.” Food Science and Technology 43: e127822. 10.1590/fst.127822.

[fsn372321-bib-0032] Yao, L. , X. Shi , H. Chen , et al. 2023. “Major Active Metabolite Characteristics of Dendrobium Officinale Rice Wine Fermented by Saccharomyces Cerevisiae and Wickerhamomyces Anomalus Cofermentation.” Food 12, no. 12: 2370. 10.3390/foods12122370. PMC10297114 37372580

[fsn372321-bib-0033] Zhang, H. W. , J. Y. Li , X. Y. Xu , et al. 2024. “Volatile Compositions and Sensorial Properties of Strawberry Fruit Wines Fermented With *Torulaspora delbrueckii* and *Saccharomyces cerevisiae* in Sequential and Simultaneous Inoculations.” European Food Research and Technology 250, no. 11: 2863–2875. 10.1007/s00217-024-04581-1.

[fsn372321-bib-0034] Zhao, H. Y. , Y. Q. Li , L. X. Liu , et al. 2022. “Effects of Inoculation Timing and Mixed Fermentation With *Pichia fermentans* on *Oenococcus oeni* Viability, Fermentation Duration and Aroma Production During Wine Malolactic Fermentation.” Food Research International 159: 111604. 10.1016/j.foodres.2022.111604. 35940798

[fsn372321-bib-0035] Zhou, W. Y. , Q. Shu , X. L. Zhang , and Q. H. Chen . 2021. “Application of Mixed‐Culture With Lactobacillus Brevis and *Saccharomyces cerevisiae* to Chinese Yellow Rice Wine Brewing for Ethyl Carbamate Regulation.” Food Control 122: 107821. 10.1016/j.foodcont.2020.107821.

