# Supplementary material for: Impact of Inoculation Strategy on Dendrobium nobile Lindl. Rice Wine Quality Through the Use of Saccharomyces cerevisiae FBKL2.8022 and Wickerhamomyces anomalus FBKL2.8023
Source: Food Sci Nutr. 2026 Sep 4;14(9):e72321. doi: 10.1002/fsn3.72321 (PMC13545422; doi:10.1002/fsn3.72321)
Supplement: Supplementary file 1 [file FSN3-14-e72321-s001.docx]

**Impact of Inoculation Strategy on *Dendrobium nobile* Lindl. rice wine Quality through the Use of *Saccharomyces cerevisiae* FBKL2.8022 and *Wickerhamomyces anomalus* FBKL2.8023**

**Authors**: Hongling Gan ^a, b, 1^, Guichun Huang ^a, b, 1^ Lin Zhang ^a, b,^ , Lanyan Cen ^a, b^ , Yifeng Dai ^a, b^ , Shuyi Qiu ^a, b^ , Xiaodan Wang ^a, b*^ , Chaoyang Wei ^a, b *^

**Affiliations**: ^a^( School of Liquor and Food Engineering, Guizhou University, Guiyang 550025, China.)

^b^(Guizhou Key Laboratory of New Quality Processing and Storage of Ecological Specialty Food, Guizhou University, Guiyang 550025, China.)

**Corresponding author**:

Chaoyang Wei, Email: cywei@gzu.edu.cn (Primary corresponding author)

Xiaodan Wang, Email: Wangxiaodan0516@126.com(Co-corresponding author)

Address correspondence to these authors at the School of Liquor and Food Engineering, Guizhou University, Guiyang 550025, China.

1**:** These authors contributed equally to this work.

## *: These authors contributed equally to this work as co-corresponding authors.

## 1. Optimization of fermentation conditions

## 1.1 Optimization of bacterial mixing ratios

Rice wine was mixed fermented using simultaneous inoculation of *Sc* and *Wa*. The operation and conditions of fermentation were controlled consistently to compare the effect of fermentation with different proportions of mixed fungi on rice wine. The ratio of *Sc*: *Wa* was set at 1:1, 5:1, 10:1, 20:1, 40:1, and 60:1.

**1.2 Optimization of the addition of *D. nobile***

Control the operation and conditions of rice wine fermentation consistently. The amount of *D. nobile* added was set at 1%, 2%, 3%, 4% and 5% based on the mass of glutinous rice.

**1.3 Optimization of the fermentation inoculation sequence**

Control of fermentation operations and conditions were consistent to compare the effect of different inoculation sequences of fermentation on rice wine. The inoculation sequence of the two yeast strains was set such that all were inoculated with *Wa* first, and *Sc* was accessed at d 0 (simultaneous inoculation of both yeast strains for fermentation), 1, 2, 3, 4, 5, and 6 of fermentation, and the fermented rice wines were recorded as Wine1, Wine2, Wine3, Wine4, Wine5, Wine6, and Wine7, respectively.

**2.** **Results of optimization of fermentation conditions**

**2.1 Proportion of** mixed fungi

All six groups of rice wines reached the fermentation end point at the 10th d (Fig. S1A), indicating that the different proportions of mixed mixed fungi bacteria had no effect on the fermentation cycle of rice wines.

When the *Sc*: *Wa* ratio was 1-10:1, the contents of total sugars, reducing sugars, total phenols, and flavonoids in rice wine increased with the increase in the percentage of *Sc*; the contents of total sugars, reducing sugars, and total phenols in rice wine reached the maximum value at the ratio of *Sc*: *Wa* 10:1; and the contents of total sugars, polysaccharides, reducing sugars, and flavonoids in rice wine decreased and then increased with the increase in the percentage of *Sc* at the ratio of *Sc*: *Wa* 10～60:1(Fig. S2). Therefore, the optimal ratio of *Sc* to *Wa* mixed fermentation was determined to be 10:1.

Fig. S1 CO_2_ weight loss diagram of rice wine fermented by mixed fungi with different ratios of *Sc* and *Wa* (A), Variation of CO_2_ weight loss of rice wine with different amounts of *D. nobile*. (B), Changes in CO_2_ weight loss of rice wine fermented with different inoculation sequences of *Sc* and *W*a mixed fungi (C) Changes in CO_2_ weight loss of rice wine (D)

Fig. S2 Physicochemical indexes of rice wine fermented with different ratios of *Sc* and *Wa*, (A) total sugar content, (B) polysaccharide content, (C) reducing sugar content, (D) total flavonoid content, (E) total phenols content.

**2.2** **Addition of** *D. nobile*

Different additions of *D. nobile* had no effect on the fermentation cycle (Fig. S1B), and there was no significant difference in the change of CO_2_ weight loss during fermentation, and all five groups of rice wines reached the end of fermentation on the 10th. *D. nobile* is rich in alkaloids. If too much *D. nobile* is added, the large amount of alkaloids dissolved in the raw material will confer a bitter flavor to the rice wine, thus affecting the taste of wine. There was no obvious characteristic aroma of dendrobium in the rice wine at 1% dendrobium addition, but the bitterness and astringency of the rice wine increased significantly with the increase of dendrobium addition. The bitter and astringent flavor of rice wine was more appropriate when *D. nobile* was added at 2% and 3%, and there was no significant effect on the taste of rice wine.

The contents of total sugars and polysaccharides in rice wine did not increase with the increase in the addition of *D. nobile*, but instead showed a trend of first increasing and then decreasing (Fig. S3A, B). The total sugar and polysaccharide content of rice wine reached the maximum value when the addition of *D. nobile* was 2%. The total phenolic and flavonoid contents in rice wine were positively correlated with the addition of *D. nobile* (Fig. S3C、D). The flavonoid and total phenol content of rice wine increased significantly and in larger increments at 2% addition of *D. nobile* compared to the previous group of rice wines. In summary, when *D. nobile* was added at 2%, it could maximally promote the dissolution of its flavonoids and phenolic active substances in the rice wine, while the rice wine had the appropriate bitterness and astringency and the best taste. Therefore, the optimum addition of *D. nobile* in rice wine was determined to be 2%.

Fig. S3 Physicochemical indexes of rice wine with different additions of *Dendrobium nobile* Lindl., (A) total sugar content, (B) polysaccharide content, (C) total flavonoid content, (D) total phenols content

**2.3** **Sequence of inoculation**

There was a significant difference in the fermentation cycle of rice wine between *Sc* and *Wa* with different inoculation sequences (Fig. S1C). The change in CO_2_ weight loss during fermentation of rice wine was found to be smaller with the time of *Sc* access to the fermentation system and the longer the fermentation period of rice wine. Wine1 and Wine2 fermented until the 10th d to reach the end of fermentation and had the greatest weight loss during rice wine fermentation. Wine5, Wine6, and Wine7 reached the end of fermentation at the 23 d, and were the three groups of rice wines with the lowest weight loss during fermentation. The weight loss during fermentation fluctuated twice, the first fluctuation was mainly due to the fermentation effect of *Wa*, while the second fluctuation was due to *Sc*, which varied with the time of its inoculation. Therefore, it was hypothesized that the overall fermentation cycle was prolonged during rice wine fermentation by inoculating *Wa* first and *Sc* from the 3rd d of fermentation probably because *Wa* became the dominant flora in the whole fermentation system. Its fermentation and metabolism products would inhibit the growth, fermentation and metabolism of *Sc*.

When *Sc* was accessed during the first 5 days of fermentation, the contents of total sugars, polysaccharides, total phenols, and flavonoids in rice wine showed an increasing trend. When *Sc* was accessed again after 5 days of fermentation, the content of reducing sugars and flavonoids gradually decreased, and the content of polysaccharides and total phenols showed a flat trend (Fig. S4). Therefore, it was determined that the optimal sequence for sequential inoculation of *Sc* and *Wa* was to inoculate *Wa* first and then access *Sc* for fermentation on the 5th d of fermentation.

Fig. S4 Physicochemical indexes of rice wine fermented in different inoculation sequences of *Sc* and *Wa*, (A) total sugar content, (B) reducing sugar content, (C) polysaccharide content, (D) total phenols content, (E) total flavonoid content

Tab. S1 Sensory evaluation of rice wine

| Indicators | 1~8 | 9~18 | 19~25 |
| --- | --- | --- | --- |
| Color and appearance | The wine has the color of the product and is cloudy. | The wine has the color of the product and is clear and transparent. | The wine has the color and lustre that this product should have, the body is clear and transparent, with luster. |
| Favor | With the unique mellow aroma of the product, no unpleasant odour | With the product's unique mellow aroma is more intense, no peculiar flavor | With the product's unique rich and mellow aroma, no peculiar flavor |
| tastes | Poor taste, slight off-flavors | Mellow and sweet, more refreshing, no off-flavors | Mellow, sweet, crisp, harmonious, no foreign flavors |
| Style | Almost no rice wine style | With a rice wine style | Typical rice wine style |

Tab. S2 Volatile substances and their contents (μg/L) in rice wine

| NO. | name | CAS | DnSW | SW | DnWS | WS |
| --- | --- | --- | --- | --- | --- | --- |
| A1 | Ethanol | 000064-17-5 | 15896.67±790.71^b^ | 19163.80±934.03^a^ | 8070.15±287.50^d^ | 10177.35±703.18^c^ |
| A2 | 1-Propanol | 000071-23-8 | － | 141.49±9.12 | － | － |
| A3 | 1-Propanol, 2-methyl- | 000078-83-1 | 195.88±11.03^b^ | 221.35±4.71^a^ | 74.70±4.55^c^ | 70.57±6.33^c^ |
| A4 | 1-Butanol | 000071-36-3 | 24.03±1.63^a^ | 24.05±2.05^a^ | 6.93±0.73^b^ | － |
| A5 | 1-Butanol, 3-methyl- | 000123-51-3 | 2900.55±163.06^a^ | 2925.71±120.79^a^ | － | － |
| A6 | 1-Pentanol | 000071-41-0 | － | － | － | 11.55±0.76 |
| A7 | 1-Pentanol, 2-methyl- | 000105-30-6 | 7.35±0.57 | － | － | － |
| A8 | 1-Hexanol | 000111-27-3 | 23.37±2.61^ab^ | － | 17.81±1.76^c^ | 20.71±0.19^bc^ |
| A9 | 1-Propanol, 3-ethoxy- | 000111-35-3 | 10.31±1.27^a^ | 9.74±1.10^a^ | － | － |
| A10 | 1-Octanol | 000111-87-5 | 32.51±4.69^b^ | 43.23±2.27^a^ | 16.20±0.16^c^ | 20.67±0.41^c^ |
| A11 | 2,3-Butanediol, | 000513-85-9 | 946.71±37.36^a^ | － | 66.61±1.98^b^ | － |
| A12 | 2,3-Butanediol, [R-(R*,R*)]- | 024347-58-8 | 432.16±41.13^b^ | 747.74±23.95^a^ | 422.44±35.01^b^ | 452.37±41.75^b^ |
| A13 | Propylene Glycol | 000057-55-6 | 44.93±2.70^a^ | 29.89±3.75^b^ | 12.06±1.35^c^ | 9.06±0.32^c^ |
| A14 | 1-Propanol, 3-(methylthio)- | 000505-10-2 | 34.65±0.29^a^ | 26.40±0.94^b^ | 13.68±0.72^c^ | 10.95±0.85^d^ |
| A15 | Phenylethyl Alcohol | 000060-12-8 | 3872.40±381.34^a^ | 3536.53±110.24^a^ | 1194.66±47.15^b^ | 1408.72±18.97^b^ |
|  | **Total Alcohols** |  | **24421.51** | **26869.92** | **9895.23** | **12181.96** |
| E1 | Ethyl Acetate | 000141-78-6 | 1008.53±18.93^c^ | 766.20±74.95^c^ | 4645.93±241.73^a^ | 3344.97±194.23^b^ |
| E2 | 1-Butanol,3-methyl-,acetate | 000123-92-2 | 419.17±72.03^a^ | 386.01±49.51^a^ | 118.92±9.35^b^ | 97.43±1.75^b^ |
| E3 | Pentanoic acid, ethyl ester | 000539-82-2 | 27.66±1.59^a^ | 16.13±1.09^b^ | 7.34±0.29^c^ | － |
| E4 | 1-Butanol, 3-methyl-, formate | 000110-45-2 | － | 25.80±1.46^c^ | 1500.56±63.68^b^ | 1721.49±94.73^a^ |
| E5 | Hexanoic acid, ethyl ester | 000123-66-0 | 366.18±25.96^b^ | 445.92±22.07^a^ | 111.63±11.35^c^ | 106.08±7.52^c^ |
| E6 | Acetic acid, hexyl ester | 000142-92-7 | 12.09±1.25^b^ | 19.81±3.22^a^ | － | － |
| NO. | name | CAS | DnSW | SW | DnWS | WS |
| E7 | Heptanoic acid, ethyl ester | 000106-30-9 | 31.35±3.58^b^ | 46.60±1.69^a^ | 12.00±1.57^d^ | 20.25±1.36^c^ |
| E8 | Propanoic acid, 2-hydroxy-, ethyl ester | 000097-64-3 | － | － | 60.56±4.68^b^ | 134.55±14.04^a^ |
| E9 | Propanoic acid, 2-hydroxy-, ethyl ester, (S)- | 000687-47-8 | － | － | 45.81±3.24^b^ | 66.84±4.21^a^ |
| E10 | Acetic acid, methoxy-, ethyl ester | 003938-96-3 | 3.41±0.90^b^ | 5.97±0.26^b^ | 35.84±3.09^a^ | － |
| E11 | Octanoic acid, ethyl ester | 000106-32-1 | 899.07±58.22^b^ | 1778.36±87.60^a^ | 312.59±46.85^c^ | 317.76±16.15^c^ |
| E12 | n-Caprylic acid isobutyl ester | 005461-06-3 | － | 22.44±0.68 | － | － |
| E13 | Butyrolactone | 000096-48-0 | 25.34±1.73^a^ | － | － | 7.68±0.24^b^ |
| E14 | Butanoic acid, 4-hydroxy- | 000591-81-1 | － | 28.01±1.50^a^ | 9.50±0.03^b^ | － |
| E15 | Decanoic acid, ethyl ester | 000110-38-3 | 715.12±58.89^b^ | 3628.57±222.70^a^ | 54.16±3.88^c^ | 60.88±1.15^c^ |
| E16 | Octanoic acid, 3-methylbutyl ester | 002035-99-6 | － | 620.47±132.18 | － | － |
| E17 | Methyl salicylate | 000119-36-8 | － | － | 6.77±0.08 | － |
| E18 | n-Capric acid isobutyl ester | 030673-38-2 | － | 34.44±3.01 | － | － |
| E19 | Acetic acid, 2-phenylethyl ester | 000103-45-7 | 426.64±30.60^b^ | 543.72±74.39^a^ | 103.85±4.31^c^ | 170.16±29.46^c^ |
| E20 | Dodecanoic acid, ethyl ester | 000106-33-2 | 672.70±80.79^b^ | 1961.19±112.04^a^ | 51.51±6.34^c^ | 123.39±10.29^c^ |
| E21 | Decanoic acid, pentyl ester | 005933-87-9 | 38.10±1.04 | － | － | － |
| E22 | Pentadecanoic acid, 3-methylbutyl ester | 002306-91-4 | 33.58±3.44^b^ | 96.90±1.24^a^ | － | － |
| E23 | Benzenepropanoic acid, ethyl ester | 002021-28-5 | － | － | 4.26±0.39 | － |
| E24 | Ethyl tridecanoate | 028267-29-0 | 25.10±0.10^a^ | 18.46±1.40^b^ | 5.08±0.28^c^ | 5.33±0.16^c^ |
| E25 | Formic acid phenyl ester | 001864-94-4 | 6.93±0.83^a^ | 7.30±1.29^a^ | － | － |
| E26 | Undecanoic acid, ethyl ester | 000627-90-7 | 50.54±4.26^b^ | 89.73±3.40^a^ | － | － |
| E27 | 2(3H)-Furanone, dihydro-5-pentyl- | 000104-61-0 | 32.40±4.78^b^ | 42.02±3.41^a^ | 33.61±2.96^b^ | 43.89±3.24^a^ |
| E28 | Tetradecanoic acid, ethyl ester | 000124-06-1 | 623.27±41.00^a^ | 685.57±58.13^a^ | 261.44±26.81^c^ | 482.90±8.29^b^ |
| E29 | Pentadecanoic acid, ethyl ester | 041114-00-5 | 24.41±3.19^a^ | 20.95±3.25^a^ | 21.01±4.03^a^ | 21.64±1.30^a^ |
| E30 | Hexadecanoic acid, ethyl ester | 000628-97-7 | 1371.85±82.26^a^ | 1384.70±233.14^a^ | 710.11±35.22^c^ | 1102.37±111.59^b^ |

| NO. | name | CAS | DnSW | SW | DnWS | WS |
| --- | --- | --- | --- | --- | --- | --- |
| E31 | Ethyl 9-hexadecenoate | 054546-22-4 | 32.84±0.33 | － | － | － |
| E32 | Ethyl Oleate | 000111-62-6 | 164.28±11.47^b^ | 135.22±12.62^b^ | 147.81±10.54^b^ | 277.89±37.93^a^ |
| E33 | Linoleic acid ethyl ester | 000544-35-4 | 352.68±48.61^a^ | 317.22±37.00^a^ | 234.29±19.64^b^ | 364.04±51.34^a^ |
|  | **Total Esters** |  | **7363.23** | **13127.71** | **8494.58** | **8469.54** |
| Al1 | Acetaldehyde | 000075-07-0 | － | 8.84±0.65 | － | － |
| Al2 | Nonanal | 000124-19-6 | 12.96±5.91^b^ | 24.55±0.24^a^ | 3.27±0.32^c^ | 14.64±1.24^b^ |
| Al3 | Benzaldehyde | 000100-52-7 | 12.05±1.16^b^ | 21.88±0.65^a^ | 21.65±1.58^a^ | － |
| Al4 | Benzeneacetaldehyde | 000122-78-1 | － | － | 4.12±0.31 | － |
| Al5 | Tetradecanal | 000124-25-4 | － | 9.29±1.17 | － | － |
|  | **Total Aldehydes** |  | **25.02** | **64.55** | **29.04** | **14.64** |
| Ac1 | Acetic acid | 000064-19-7 | 180.48±19.07^d^ | 1552.24±62.11^a^ | 447.98±17.74^c^ | 716.67±35.24^b^ |
| Ac2 | Propanoic acid, 2-methyl- | 000079-31-2 | 22.33±2.09 | － | － | － |
| Ac3 | 2-Amino-6-methylbenzoic acid | 004389-50-8 | 64.02±3.69^a^ | － | 24.16±2.50^b^ | － |
| Ac4 | 2-Amino-5-methylbenzoic acid | 002941-78-8 | － | 34.15±0.36 | － | － |
| Ac5 | Neodecanoic acid | 026896-20-8 | 148.05±4.60^a^ | 71.61±0.75^b^ | 25.18±1.60^c^ | － |
| Ac6 | Heptanoic acid | 000111-14-8 | － | － | 17.36±0.52^a^ | 4.50±0.09^b^ |
| Ac7 | Octanoic acid | 000124-07-2 | 76.71±8.25^a^ | 77.17±4.95^a^ | 21.19±0.73^b^ | 19.67±0.71^b^ |
| Ac8 | Nonanoic acid | 000112-05-0 | － | － | － | 6.15±0.06 |
|  | **Total Acids** |  | **491.60** | **1735.16** | **535.85** | **746.99** |
| KP1 | 2-Octanone | 000111-13-7 | 52.33±4.15^a^ | 44.62±4.53^ab^ | 37.84±5.06^b^ | 42.47±2.74^b^ |
| KP2 | Acetoin | 000513-86-0 | 52.10±2.02^c^ | 41.93±2.62^d^ | 58.02±2.91^b^ | 88.85±0.33^a^ |
| KP3 | Eugenol | 000097-53-0 | － | － | 4.78±0.16 | － |
| KP4 | Butylated Hydroxytoluene | 000128-37-0 | － | － | － | 12.86±0.56 |
| KP5 | 2-Methoxy-4-vinylphenol | 007786-61-0 | 79.22±0.50^d^ | 109.71±11.77^c^ | 194.88±16.28^b^ | 257.34±23.92^a^ |

| NO. | name | CAS | DnSW | SW | DnWS | WS |
| --- | --- | --- | --- | --- | --- | --- |
|  | **Total Ketones and Phenols** |  | **183.65** | **196.25** | **295.52** | **401.52** |
| O1 | .beta.-Ocimene | 013877-91-3 | 11.15±0.86^a^ | － | 6.07±0.62^b^ | － |
| O2 | Styrene | 000100-42-5 | 22.09±2.52^b^ | 36.01±0.16^a^ | 13.32±0.63^c^ | 15.53±0.82^c^ |
| O3 | Pentadecane | 000629-62-9 | 68.20±3.64^b^ | 104.87±7.28^a^ | 42.71±3.79^c^ | 48.95±5.70^c^ |
| O4 | Benzene, 1,4-diethyl- | 000105-05-5 | 7.51±0.38^b^ | 14.44±1.06^a^ | 7.92±0.52^b^ | 6.76±0.42^b^ |
| O5 | Benzene, pentyl- | 000538-68-1 | 4.15±0.61^a^ | 4.72±0.11^a^ | 0.00 | 2.95±0.40^b^ |
| O6 | Naphthalene | 000091-20-3 | － | － | 32.90±1.34^a^ | 25.04±2.59^b^ |
| O7 | 1,2,3,4-Butanetetrol, [S-(R*,R*)]- | 002319-57-5 | 11.17±1.12^a^ | 3.84±0.56^c^ | 7.51±0.12^b^ | － |
| O8 | Benzofuran, 2,3-dihydro- | 000496-16-2 | 14.55±1.28^a^ | 18.90±0.50^a^ | 8.08±5.15^b^ | 14.08±0.09^a^ |
| O9 | 2-Phenylindolizine | 025379-20-8 | 20.10±1.04 | － | － | － |
|  | **Total Others** |  | **158.93** | **182.77** | **118.51** | **113.32** |
|  | **Totals** |  | **32485.00** | **41993.60** | **19250.22** | **21814.66** |

Note: 1. ‘-’ indicates not detected; 2. Different lowercase letters indicate significant differences, P<0.05.

**Tab.S3 Relative percentage of major active metabolites in rice wine**

| NO. | Compounds | Classification | Mode | SW | WS | DnSW | DnWS |
| --- | --- | --- | --- | --- | --- | --- | --- |
| 1 | 3-(3,4,5-Trimethoxyphenyl)propanoic acid | Phenylpropanoids | P | 0.12 | 0.34 | 0.11 | 0.64 |
| 2 | Methyldopa | Phenylpropanoids | P | 0.02 | 0.01 | 0.06 | 0.04 |
| 3 | Licochalcone B | Phenylpropanoids | P | 0.01 | 0.01 | 0.02 | 0.03 |
| 4 | 2-Hydroxy-3-(4-methoxyphenyl)propanoic acid | Phenylpropanoids | P | 0.01 | 0.01 | 0.05 | 0.04 |
| 5 | Zeranol | Phenylpropanoids | P | － | － | 0.01 | 0.01 |
| 6 | Aspalathin | Phenylpropanoids | N | － | － | 0.23 | － |
| 7 | Capsaicin | phenolic | P | － | － | 4.76 | 5.59 |
| 8 | 4-Hydroxy-2,6-dimethylaniline | phenolic | P | 1.55 | 5.27 | 1.53 | 10.06 |
| 9 | Acetaminophen | phenolic | N | 1.29 | 1.01 | 1.44 | 1.01 |
| 10 | Nordihydrocapsaicin | phenolic | P | － | － | 1.19 | 1.41 |
| 11 | o-Cresol | phenolic | N | 0.91 | 0.50 | 0.98 | 0.47 |
| 12 | Hydroxytyrosol | phenolic | N | 0.35 | 0.23 | 0.64 | 0.54 |
| 13 | Phenol | phenolic | P | 0.15 | 0.07 | 0.23 | 0.20 |
| 14 | 3,4-Dihydroxyphenylvaleric acid | phenolic | P | － | － | 0.04 | 0.05 |
| 15 | Epicatechin | flavonoids | N | 0.05 | 0.13 | 0.04 | 0.11 |
| NO. | Compounds | Classification | Mode | SW | WS | DnSW | DnWS |
| 16 | Mulberrin | flavonoids | N | － | － | 0.23 | 0.17 |
| 17 | Sakuranetin | flavonoids | N | 0.01 | 0.05 | 0.01 | 0.13 |
| 18 | (+)-Gallocatechin | flavonoids | N | 0.25 | 0.35 | 0.24 | 0.18 |
| 19 | Myricetin | flavonoids | N | 0.17 | 0.18 | 0.15 | 0.19 |
| 20 | Heterophyllin | flavonoids | P | 0.10 | 0.13 | 0.09 | 0.11 |
| 21 | Miscanthoside | flavonoids | N | 0.25 | 0.16 | 0.15 | 0.08 |
| 22 | Quercetin | flavonoids | N | 0.06 | 0.07 | 0.06 | 0.08 |
| 23 | Kaempferol 3-alpha-L-arabinofuranoside | flavonoids | P | 0.01 | 0.02 | 0.02 | 0.02 |
| 24 | Isoginkgetin | flavonoids | P | 0.01 | 0.01 | 0.02 | 0.02 |
| 25 | Herbacetin 3,8-diglucoside | flavonoids | P | － | － | － | 0.01 |
| 26 | Licorisoflavan A | flavonoids | P | 0.09 | 0.09 | 0.01 | 0.02 |
| 27 | Formononetin 7-O-glucuronide | flavonoids | P | － | － | 0.01 | 0.01 |
| 28 | Hesperidin | flavonoids | P | 0.01 | － | － | － |
| 29 | 5-hydroxy-7-methoxy-2-(4-methoxyphenyl)-4H-chromen-4-one | flavonoids | P | 0.03 | － | 0.03 | － |
| 30 | trans-Cinnamic acid | lignin | N | 13.05 | 74.76 | 15.73 | 161.11 |
| 31 | trans-Ferulic acid | lignin | N | 20.61 | 21.81 | 17.52 | 15.22 |
| NO. | Compounds | Classification | Mode | SW | WS | DnSW | DnWS |
| 32 | 2-Benzylidene-1-heptanol | lignin | P | － | － | 1.93 | 1.90 |
| 33 | Sinapic acid | lignin | N | 0.45 | 0.94 | 0.96 | 4.56 |
| 34 | 2-Hydroxycinnamic acid | lignin | N | 1.93 | 1.32 | 2.42 | 1.85 |
| 35 | Subaphylline | lignin | P | 0.15 | 0.98 | 0.13 | 0.90 |
| 36 | (7'R)-(+)-Lyoniresinol 9'-glucoside | lignin | N | － | － | 0.01 | 0.14 |
| 37 | 4-O-Caffeoylshikimic acid | lignin | P | － | 0.01 | 0.04 | 0.03 |
| 38 | Coniferin | lignin | P | － | － | 0.05 | 0.06 |
| 39 | 8-Acetoxypinoresinol | lignin | P | － | － | 0.01 | 0.01 |
| 40 | (Z)-N-Feruloyl-5-hydroxyanthranilic acid | lignin | P | 0.01 | 0.01 | 0.01 | 0.01 |
| 41 | Adenine | alkaloid | N | 181.18 | 181.91 | 191.49 | 191.32 |
| 42 | Xanthine | alkaloid | N | 52.81 | 53.85 | 53.08 | 48.94 |
| 43 | Theophylline | alkaloid | N | 0.88 | 1.41 | 1.07 | 1.74 |
| 44 | Piperidine | alkaloid | P | 0.39 | 2.26 | 0.41 | 3.60 |
| 45 | Indoleacetaldehyde | alkaloid | N | 0.57 | 4.04 | 0.67 | 3.48 |
| 46 | Calystegine B2 | alkaloid | P | 0.30 | 0.38 | 0.27 | 0.96 |
| 47 | Harman | alkaloid | P | 0.11 | 1.06 | 0.06 | 1.04 |
| NO. | Compounds | Classification | Mode | SW | WS | DnSW | DnWS |
| 48 | Porphobilinogen | alkaloid | N | 0.09 | 0.33 | 0.09 | 0.53 |
| 49 | Indole | alkaloid | P | 0.08 | 0.32 | 0.08 | 0.36 |
| 50 | 1-Deoxynojirimycin | alkaloid | P | 0.18 | 0.16 | 0.19 | 0.18 |
| 51 | Trigonelline | alkaloid | P | 0.12 | 0.07 | 0.16 | 0.16 |
| 52 | Norsanguinarine | alkaloid | P | 0.03 | 0.09 | 0.05 | 0.13 |
| 53 | 1,3,7-Trimethyluric acid | alkaloid | N | 0.14 | 0.13 | 0.18 | 0.08 |
| 54 | xi-2,3-Dihydro-2-oxo-1H-indole-3-acetic acid | alkaloid | N | 0.05 | 0.10 | 0.06 | 0.08 |
| 55 | 2,4-Dimethyl-1H-indole | alkaloid | P | 0.06 | 0.22 | 0.07 | 0.63 |
| 56 | Histidinal | alkaloid | P | 0.07 | 0.07 | 0.06 | 0.07 |
| 57 | 3-Isoxazolidinone | alkaloid | P | 0.01 | 0.04 | 0.01 | 0.08 |
| 58 | Imidazole-4-acetaldehyde | alkaloid | P | 0.10 | 0.05 | 0.09 | 0.06 |
| 59 | Imidazoleacetic acid | alkaloid | P | 0.93 | 0.27 | 1.23 | 0.22 |
| 60 | L-Histidinol | alkaloid | P | 0.94 | 0.89 | 0.76 | 0.85 |
| 61 | Temurin | alkaloid | P | 0.05 | 0.06 | 0.05 | 0.05 |
| 62 | Caffeine | alkaloid | P | 0.05 | 0.03 | 0.05 | 0.03 |
| 63 | Arecaidine | alkaloid | P | 0.05 | 0.03 | 0.07 | 0.04 |
| NO. | Compounds | Classification | Mode | SW | WS | DnSW | DnWS |
| 64 | Betanidin | alkaloid | P | 0.06 | 0.05 | 0.04 | 0.04 |
| 65 | Betaine | alkaloid | P | 0.02 | 0.03 | 0.03 | 0.04 |
| 66 | 6-Methylquinoline | alkaloid | P | 0.08 | 0.03 | 0.06 | 0.03 |
| 67 | 6-Hydroxy-1H-indole-3-acetamide | alkaloid | P | 0.01 | 0.01 | 0.02 | 0.03 |
| 68 | Sphinganine | alkaloid | P | 0.07 | 0.05 | 0.06 | 0.03 |
| 69 | Pyrroloquinoline quinone | alkaloid | P | 0.04 | 0.03 | 0.04 | 0.04 |
| 70 | 2,2,6,6-Tetramethyl-4-piperidinone | alkaloid | P | 0.02 | 0.02 | 0.02 | 0.02 |
| 71 | Fagomine | alkaloid | P | 0.00 | 0.12 | 0.00 | 0.11 |
| 72 | Deuteroporphyrin IX | alkaloid | P | 0.00 | 0.00 | 0.00 | 0.00 |
| 73 | Genipin | terpenoid | N | 0.12 | 0.49 | 0.14 | 2.13 |
| 74 | Thymol methyl ether | terpenoid | P | 0.67 | 0.89 | 1.66 | 1.79 |
| 75 | Dihydroisoalantolactone | terpenoid | P | 0.00 | 0.00 | 1.03 | 1.10 |
| 76 | beta-Costic acid | terpenoid | P | 0.00 | 0.00 | 0.69 | 0.71 |
| 77 | Alantolactone | terpenoid | P | 0.00 | 0.00 | 0.35 | 0.39 |
| 78 | Spathulenol | terpenoid | P | 0.00 | 0.00 | 0.31 | 0.37 |
| 79 | Erinacine C | terpenoid | P | 0.36 | 0.37 | 0.37 | 0.29 |
| NO. | Compounds | Classification | Mode | SW | WS | DnSW | DnWS |
| 80 | Xanthorrhizol | terpenoid | P | － | － | 0.19 | 0.28 |
| 81 | Valerenolic acid | terpenoid | P | － | － | 0.31 | 0.34 |
| 82 | Abscisic acid | terpenoid | N | － | 0.11 | 0.06 | 0.19 |
| 83 | 1,4-Dimethyl-7-ethylazulene | terpenoid | P | 0.03 | 0.01 | 0.18 | 0.18 |
| 84 | 3,4-Dihydrocadalene | terpenoid | P | － | － | 0.13 | 0.15 |
| 85 | Perillyl aldehyde | terpenoid | P | 0.03 | 0.04 | 0.11 | 0.15 |
| 86 | Valerenal | terpenoid | P | － | － | 0.09 | 0.10 |
| 87 | Erinacine P | terpenoid | P | 0.08 | 0.11 | 0.06 | 0.09 |
| 88 | Capsidiol | terpenoid | P | － | － | 0.09 | 0.08 |
| 89 | Cinncassiol A 19-glucoside | terpenoid | P | 0.02 | 0.05 | 0.02 | 0.02 |
| 90 | Cinncassiol A | terpenoid | P | 0.03 | 0.05 | 0.03 | 0.08 |
| 91 | Demethyloleuropein | terpenoid | P | － | － | 0.05 | 0.05 |
| 92 | Ginkgolide J | terpenoid | N | 0.01 | 0.13 | 0.01 | 0.04 |
| 93 | Obacunone | terpenoid | P | － | － | 0.03 | 0.03 |
| 94 | Cinnzeylanol | terpenoid | P | 0.03 | 0.02 | 0.02 | 0.04 |
| 95 | Dihydrofukinolide | terpenoid | P | 0.02 | 0.02 | 0.02 | 0.01 |
| NO. | Compounds | Classification | Mode | SW | WS | DnSW | DnWS |
| 96 | Ginkgolide C | terpenoid | P | － | － | 0.01 | 0.01 |
| 97 | Geniposidic acid | terpenoid | P | － | － | 0.02 | 0.01 |
| 98 | Valtrate | terpenoid | P | 0.01 | 0.01 | 0.01 | 0.01 |
| 99 | Dihydrovaltrate | terpenoid | P | 0.01 | 0.01 | 0.01 | 0.01 |
| 101 | Dexamethasone | Steroids | P | 0.29 | 0.81 | 0.22 | 0.52 |
| 102 | Jangomolide | Steroids | P | － | － | 0.10 | 0.11 |
| 103 | Nandrolone | Steroids | P | 0.05 | 0.07 | 0.04 | 0.09 |
| 104 | Dehydroisoandrosterone 3-glucuronide | Steroids | P | 0.06 | 0.05 | 0.05 | 0.06 |
| 105 | Withaperuvin D | Steroids | P | 0.07 | 0.03 | 0.05 | 0.05 |
| 106 | Androsterone sulfate | Steroids | P | 0.02 | 0.02 | 0.01 | 0.03 |
| 107 | Physalolactone | Steroids | P | 0.03 | 0.02 | 0.03 | 0.02 |
| 108 | Stigmasterol | Steroids | P | － | － | 0.01 | 0.01 |
| 109 | Estriol | Steroids | P | 0.01 | 0.01 | 0.01 | 0.01 |
| 110 | 5a-Tetrahydrocortisol | Steroids | P | 0.03 | 0.01 | 0.01 | 0.01 |
| 111 | Clionasterol | Steroids | P | － | 0.01 | － | 0.01 |

Note: “-” in the table indicates that the relative percentage content of the substance in rice wine is less than 0.01.
